# A Site‐Aware Representation Learning Framework For Unified Molecular Interaction Modeling and Generative Design

**DOI:** 10.1002/advs.77238

**Published:** 2026-09-10

**Authors:** Gang Luo, Qianqian Zhang, Chenhao Wang, Zhiheng Yi, Alex Jinpeng Wang, Jing Tang, Min Li

**Affiliations:** ^1^ School of Computer Science and Engineering Central South University Changsha China; ^2^ Sai Ling Pharmaceutical Technology Group Co., Ltd. Yuxi China; ^3^ Research Program in Systems Oncology Faculty of Medicine University of Helsinki Helsinki Finland

**Keywords:** binding‐site prediction, deep learning, drug‐target interactions, intrinsically disordered proteins, molecular design

## Abstract

Unifying drug‐target affinity prediction and targeted molecular design within a single interpretable framework remains challenging. Many sequence‐based affinity and design methods rely on global target representations without explicitly modeling binding regions, leading to site‐level ambiguity in both screening and design. By contrast, structure‐based methods require high‐quality structural data and are poorly suited to dynamic targets. In this study, a new model named MolDBG is proposed as a unified site‐aware framework that combines affinity prediction, binding‐site identification, and affinity‐conditioned molecular generation within a single architecture. With binding‐site supervision, MolDBG prioritizes interaction‐critical residues before learning drug‐target representations, reducing false positives from misaligned binding sites and improving interpretability. MolDBG achieves competitive performance across all three tasks while enabling site‐specific affinity prediction and interpretable binding‐site discovery. The framework generalizes to structurally elusive targets, including cryptic pockets and intrinsically disordered proteins. Overall, these results demonstrate that MolDBG is a promising framework for molecular design and screening.

## Graphical abstract

MolDBG is a site‐aware, sequence‐only framework that unifies drug‐target affinity prediction, binding‐site identification, and affinity‐conditioned molecular generation for structured proteins. Guided by multi‐task binding‐site supervision, it aligns interaction‐critical residues before learning drug‐target representations and simultaneously infers binding sites alongside affinity prediction and molecule generation to improve interpretability. It further extends binding‐site prediction to cryptic pockets and intrinsically disordered proteins.

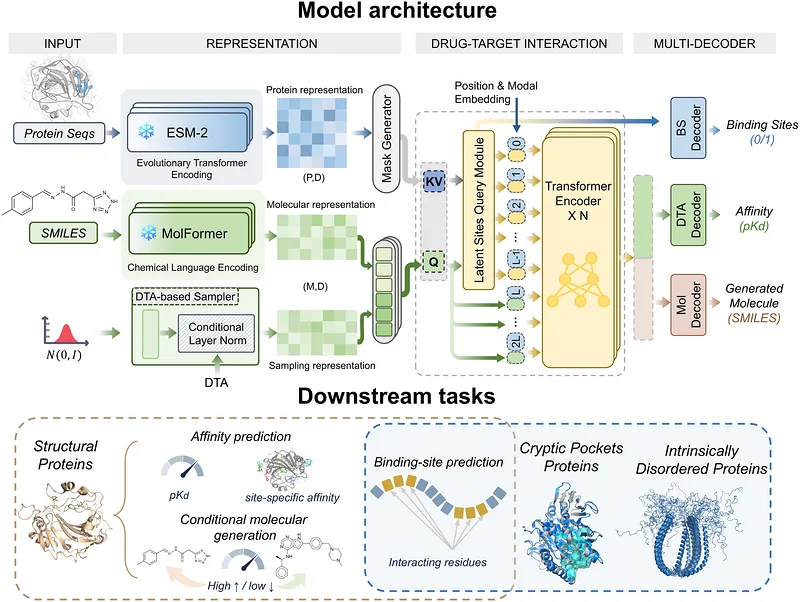

## 1 Introduction

Deep learning has precipitated significant advancements in drug discovery. Previous studies have developed deep learning architectures applied across the entire drug development pipeline, ranging from de novo molecular design [1, 2] and targeted molecular design [3, 4, 5] to property prediction [6, 7] and drug‐target interaction (DTI) prediction [8, 9, 10, 11]. The drug discovery paradigm typically follows a design‐then‐screen pipeline, in which molecules are designed de novo and their drug‐target affinity (DTA) is accurately predicted. For the DTA prediction task, previous studies have used Convolutional Neural Networks (CNN) [12, 13], Transformers [14], and Graph Neural Networks (GNN) [9, 15] to extract features from molecules and protein sequences, achieving stable affinity predictions. With the rise of representation learning [16] and large language models [17], such architectures have naturally transitioned into the life sciences [18, 19]. By acquiring highly generalizable semantic representations, they have significantly enhanced performance in downstream tasks such as protein function prediction [20], active site prediction [21], and molecular property prediction [22]. Furthermore, these pre‐trained representations [23, 24, 25, 26] are also widely used to augment DTA prediction [27, 28].

Although affinity prediction models have achieved high precision, the drug discovery pipeline remains constrained to the generation of molecules followed by DTA‐based high‐throughput screening. Although the reinforcement learning methods [29, 30, 31, 32] enables the one‐step generation of targeted small molecules with specific activities, it remains constrained to a limited set of target proteins. Meanwhile, the structure‐based generation models [33, 34, 35] demonstrate the potential for targeted high‐affinity small‐molecule design based on the target protein's 3D structural information, but these approaches rely heavily on high‐quality protein structural data. DeepDTAGen [36] integrates drug‐target binding affinity and conditional molecular generation tasks without protein structure, which provides a route for high‐affinity conditional molecular design. However, it is hard to address the site‐level affinity prediction and lacks interpretability.

Despite extensive efforts that have been devoted to AI‐aided drug discovery, there are still several limitations in the previous studies. First, there may be spatial mismatches between the target‐aware affinity and the molecule‐generation methods. Many natural proteins possess multiple pockets [37] or allosteric sites [38], and the target‐drug spatial mismatch leads to frequent false positives or failed targeted molecular design due to misaligned binding sites. Second, existing DTA prediction and targeted design methods often lack interpretability because they rely heavily on physicochemical features or pre‐trained representations. Although some studies [39] have visualized the attention weights to analyze key residues in binding regions, they lack explicit binding site evaluation and outputs. Third, the existing methods are insufficient for dynamic structure protein‐related affinity prediction and molecule de novo design. The structure‐based models rely heavily on high‐quality crystal data. Although the development of protein structure prediction models [40, 41] has expanded the scope of related methods [42], there remain significant limitations for cryptic pocket proteins [43] and intrinsically disordered proteins (IDPs) [44].

Affinity prediction and conditional molecular design are fundamentally grounded in the analysis of protein‐ligand interactions within specific interaction regions and share an intrinsic modeling correlation centered on binding sites. In this study, we propose a unified site‐aware framework, MolDBG, for molecule‐centric drug‐target affinity prediction, binding site identification, and conditional molecule generation. Specifically, MolDBG employs a sequence‐only modeling strategy that enables DTA prediction and affinity‐conditional design without explicit protein structural data. By incorporating the binding site masking strategy and site prediction task, our method achieves robust performance in distinguishing binding affinities for ligand‐protein interactions across different binding sites within the same protein. It effectively mitigates false positives associated with global target‐aware predictions and endows the model with high interpretability. Experimental evaluations indicate that our approach consistently outperforms baselines across all three base tasks. Our model also shows strong generalization in site‐level affinity prediction. Furthermore, we conducted extensive binding site prediction experiments on the cryptic pocket proteins (PocketMiner) and the IDPs (UniProtKB‐IDP) independent tests, and achieved an AUROC of 0.9014 on PocketMiner and a sample‐level success rate of 72.62% on UniProtKB‐IDP. In summary, these experiments demonstrate the efficacy and flexibility of MolDBG across the entire drug discovery pipeline.

## 2 Results

### 2.1 Overview of MolDBG

**FIGURE 1:**
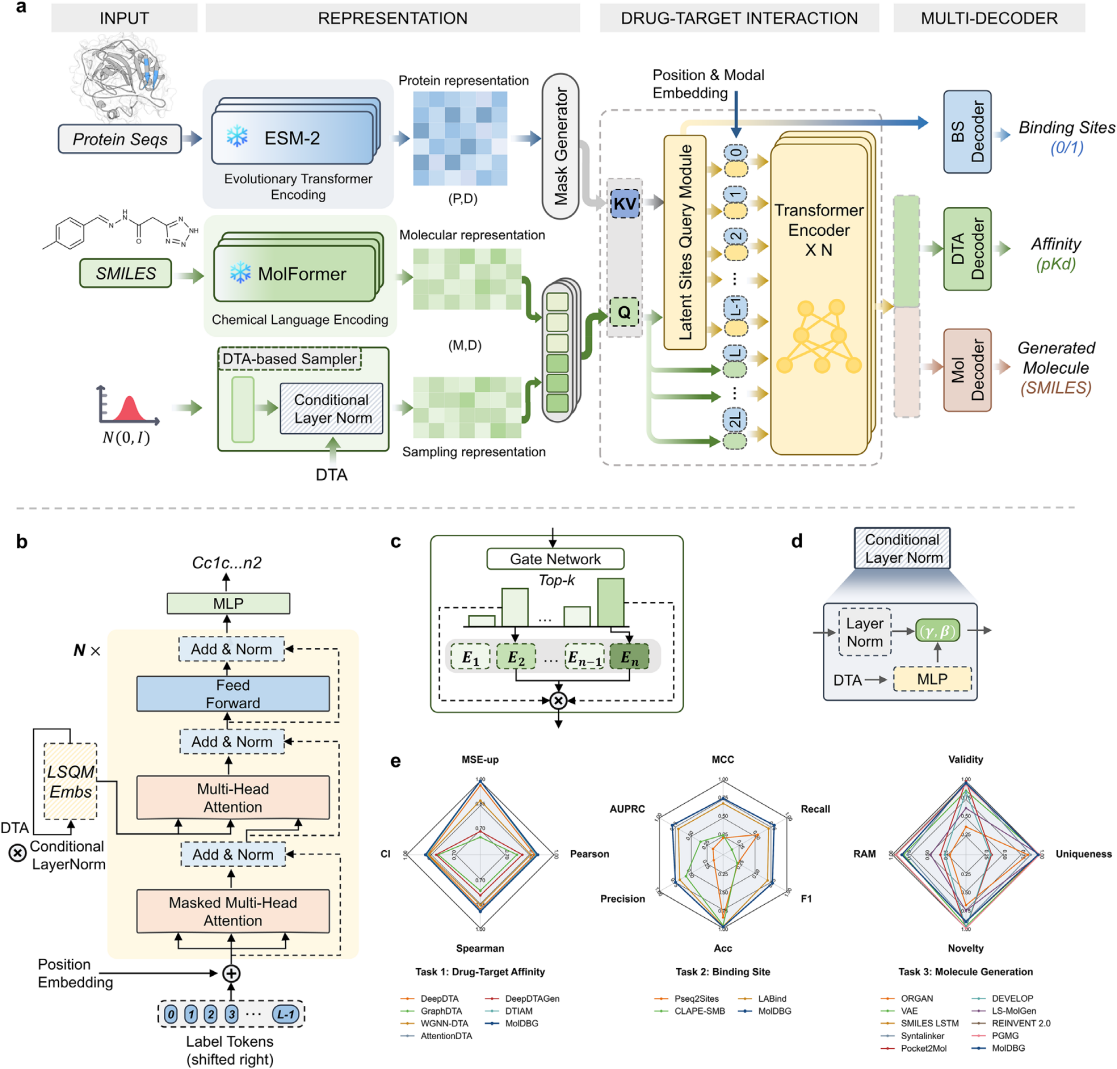
Overview of the MolDBG framework and performance benchmarks. (a) The MolDBG architecture. ESM‐2 and MolFormer serve as the pre‐trained protein and molecular encoders, respectively, extracting token‐level representations directly from sequence inputs. N(0,I) denotes a random tensor sampled from a standard normal distribution. These outputs are processed to construct protein representations (blue tensors) and molecular representations (green tensors), which function as Key/Value (KV) and Query (Q) inputs for the Latent Sites Query Module (LSQM). Finally, the fused representations are split along the batch dimension to facilitate affinity prediction and molecular design via distinct decoders. The Binding Sites Decoder operates directly on LSQM query results. (b) The conditional molecular decoder module. (c) The Mixture of Experts within the Drug‐Target Affinity Decoder. (d) Design of the Conditional LayerNorm module utilized in both the DTA‐based sampler and the Molecular Decoder. (e) Radar chart comparison across three primary tasks. Standard evaluation metrics are displayed, with model legends provided at the bottom. To facilitate visualization of the Mean Squared Error, we defined an “MSE‐up” metric. The baseline model with the highest (worst) MSE was set as the reference base (normalized to 0.5), and the relative percentage improvement of other models over this base was calculated for plotting.

MolDBG constitutes a multi‐task, unified learning framework centered on binding site identification. The insight of our approach is that both affinity prediction and conditional molecular design fundamentally involve modeling interactions between specific protein sites and small molecules under physical binding‐interaction constraints. The overall architecture of MolDBG is illustrated in Figure 1a. The primary objective of MolDBG is to simultaneously model affinity prediction and conditional molecule generation, while identifying ligand‐protein binding sites, all without reliance on 3D structural data. Initially, our model input representations are obtained via the respective molecular and protein language models. Specifically, molecular SMILES strings and protein sequences are processed as text inputs. We employ the pre‐trained models ESM‐2 and MolFormer as protein and molecule encoders, respectively, to derive token‐level representations. Notably, for the molecular design task, we devised an affinity‐based sampler that randomly samples molecules conditioned on affinity (Figure 1d) to generate a pseudo‐ligand representation. Subsequently, using the molecular representation as the Query and the protein representation as both Key and Value, we construct an attention‐driven LSQM module to extract representations of potential binding residue tokens. We introduce a binding site prediction task to constrain the LSQM module. We apply explicit supervised learning to the LSQM module via this binding‐site prediction task and reinforce the transition from attention‐driven querying to physical‐binding interaction querying. Following the injection of modality and positional encoding information into the molecular and latent binding token representations, we design a multi‐layer architecture employing self‐attention mechanisms to establish cross‐modal correlations and construct a binding representation space corresponding to the drug‐target interaction region. The MolDBG framework comprises two primary decode pipelines: molecular design and affinity prediction. For conditional molecular design, the affinity‐infused binding representation (derived from the pseudo‐ligand branch) serves as the drug‐target interaction context. An autoregressive Transformer decoder is then employed to generate SMILES tokens sequentially, as shown in Figure 1b, thereby ensuring the generation of structurally valid molecules with targeted activity; For drug‐target affinity prediction, we design a regression module incorporating a simple feature aggregation layer and a Mixture of Experts (MoE) module (Figure 1c). This affinity prediction module performs non‐linear fitting on the binding representation (derived from the real‐ligand branch) to predict affinity values. The entire model is optimized end‐to‐end, promoting the sharing of interaction representations across tasks within the intermediate fusion layers. As shown in Figure 1e, the experimental results demonstrate that our unified model achieves performance comparable to domain‐specific representative baselines across nearly all evaluation metrics.

### 2.2 Drug‐Target affinity Prediction Performance

**FIGURE 2:**
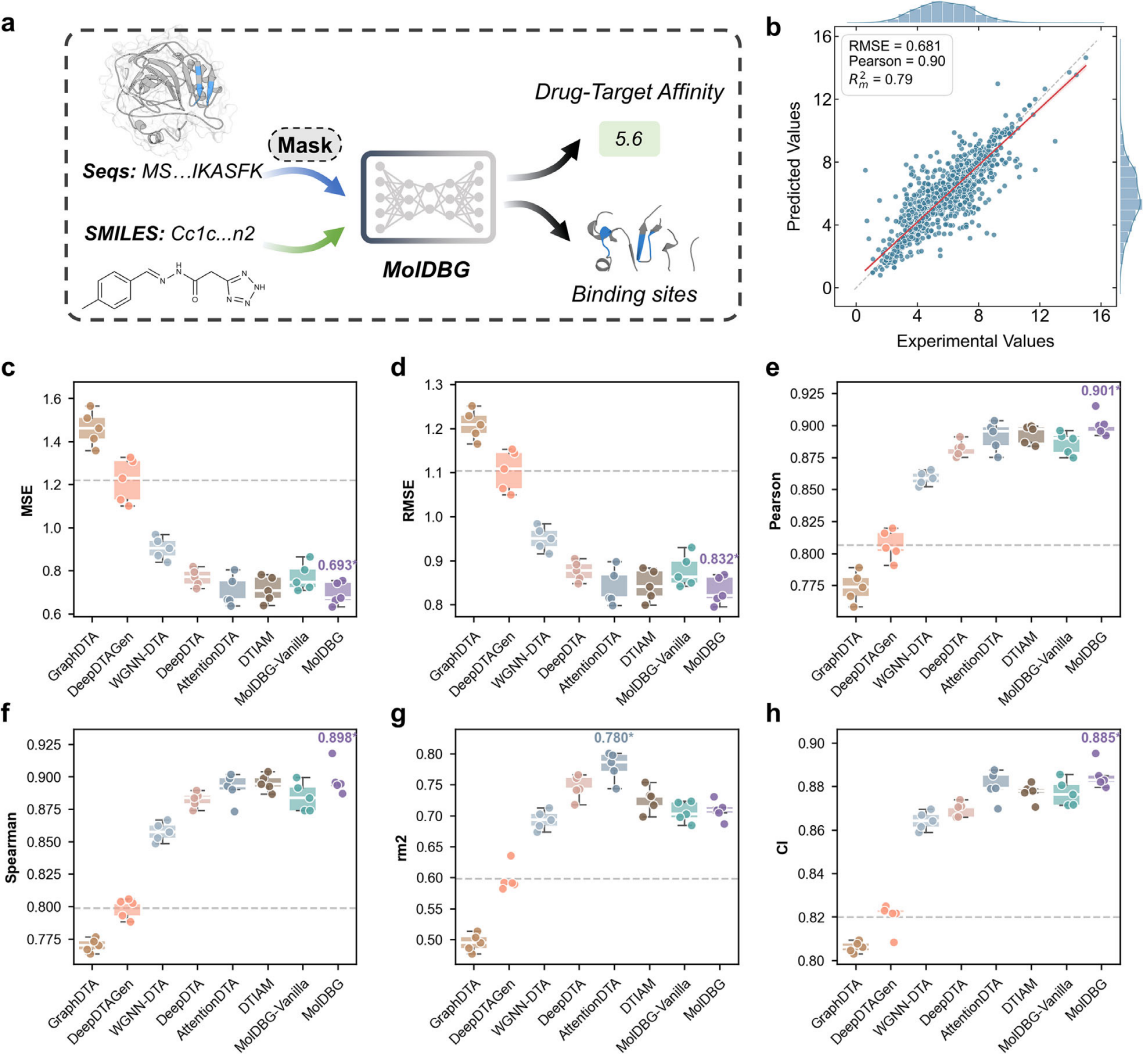
Workflows and performance evaluation for drug‐target affinity prediction. (a) Pipelines for drug‐target affinity prediction, distinguishing between full protein‐based and specific binding site‐based input modalities. (b) Scatter plots illustrating the correlation between predicted affinity values and experimentally measured affinities across the held‐out validation folds. (c–h) Comparison results of affinity prediction task models. Box plots illustrating the performance distribution of DTA prediction task across six evaluation metrics: (c) MSE, (d) RMSE, (e) Pearson, (f) Spearman, (g) $r_{m}^{2}$, and (h) CI. All models were evaluated using 5‐fold cross‐validation on the BioLip‐BS dataset. The scatter points overlaid on the boxes represent the individual results from the 5‐fold cross‐validation. The numerical values highlighted above the bars indicate the mean performance of the best‐performing model for each metric. The horizontal gray dashed lines denote the mean of DeepDTAGen.

In the DTA prediction task, we benchmarked our framework against six baseline models: DeepDTA [12], GraphDTA [15], WGNN‐DTA [45], AttentionDTA [14], DeepDTAGen [36], and DTIAM [11]. All baseline models are retrained and evaluated on the BioLip‐BS dataset. The results are presented in Figure 2c–h, and the comparison between predicted affinities and ground truth labels on the held‐out validation folds is illustrated in Figure 2b. MolDBG achieved competitive performance in the Mean Squared Error (MSE), Pearson correlation coefficient (Pearson), and Concordance Index (CI), with values of 0.6933, 0.9009, and 0.8850, respectively. Notably, MolDBG matched the high CI value of DTIAM (0.8850), indicating exceptional consistency in prediction rankings. The advantages of our approach are particularly pronounced compared to early graph neural network architectures such as GraphDTA, with MolDBG achieving a 52.3% reduction in MSE and an approximate 16.4% improvement in Pearson. Furthermore, the MolDBG model reduced the MSE by 0.0762 (10%) relative to MolDBG‐Vanilla (non‐pretrained) while achieving a higher correlation coefficient, thereby substantiating the efficacy of incorporating pre‐training strategies and binding‐site prediction tasks for accurately modeling interaction mechanisms. Additionally, MolDBG showed consistent improvements in metrics such as MSE and Pearson coefficient compared to DeepDTAGen, a model designed for similar tasks. This underscores the necessity of synergizing large‐scale pre‐trained representations with task‐specific adaptation to ensure robust affinity prediction. We attribute the comparatively lower performance of DeepDTAGen to the limited size of the BioLip‐BS dataset, which complicates the direct construction of molecular design models and impedes gradient convergence within a dual‐task framework. In addition, to rigorously evaluate the generalization capability of MolDBG in realistic drug discovery scenarios, we performed a strict sequence‐identity‐based cold‐start evaluation on the BioLip‐BS dataset. We present these results in the Section S5 and Table S4. MolDBG consistently outperforms DTIAM across all ranking and correlation metrics under protein and molecule cold‐start settings. These results demonstrate that MolDBG generalizes to unseen target proteins and ligands by capturing structural binding patterns rather than relying on sequence memorization. Collectively, these results demonstrate that the multi‐task synergistic modeling employed by MolDBG enhances both prediction accuracy and generalization in drug‐target affinity tasks.

### 2.3 Site‐Specific Affinity Prediction and the Discrimination of Regional Binding Variations

Conventional target‐aware methods often fail to account for variations in binding affinity across distinct binding sites. While structure‐based approaches have offered improvements, they remain constrained by their reliance on high‐quality structural data, limiting their general applicability. To address this, we implemented a perturbation window strategy within the Latent Sites Query Module (LSQM) of MolDBG during fine‐tuning. By stochastically alternating between full‐protein representations and restricted potential binding regions within the same training batch, we enabled the model to perform affinity prediction and molecular design conditioned on specific local environments. This approach effectively resolves the inability of traditional target‐aware models to differentiate the binding strengths of a single molecule across multiple pockets.

**FIGURE 3:**
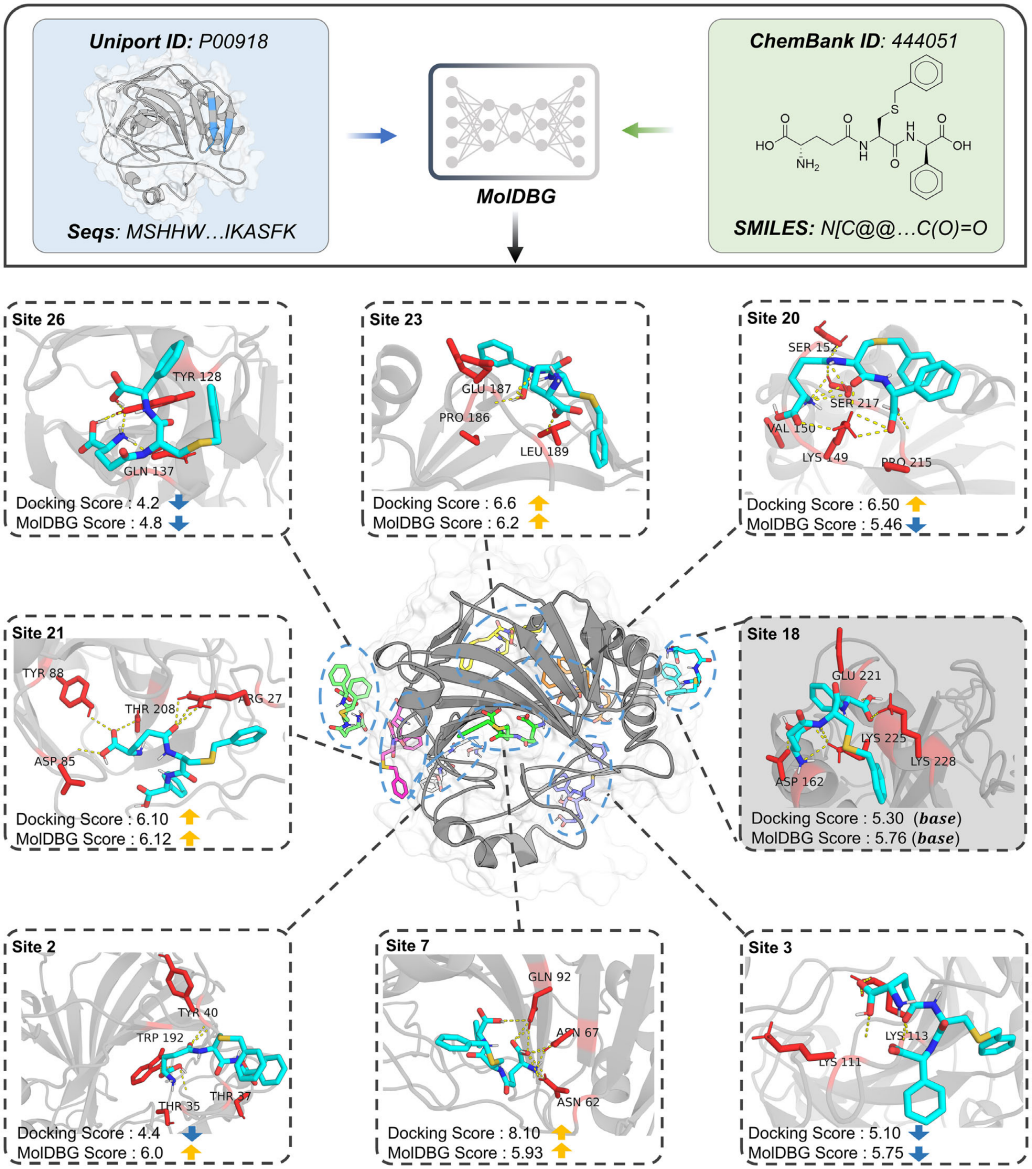
Comparison of site‐specific affinity predictions by MolDBG and AutoDock Vina. The panel illustrates variations in binding strength for identical molecules across distinct binding regions. Site‐18 serves as the baseline reference for interaction strength; ↑ indicates an increase relative to the baseline, while ↓ denotes a decrease. Docking scores shown here are the negated AutoDock Vina scores (i.e., ‐Vina), so that larger values indicate stronger predicted binding, aligning the direction with MolDBG affinity predictions.

We selected Carbonic Anhydrase II (CA2; UniProt ID: P00918) as a case study for validation, which is widely expressed in erythrocytes, kidneys, eyes, and the central nervous system, playing a critical role in acid‐base homeostasis, electrolyte transport, and intraocular pressure regulation. We leveraged binding site annotations from the UniSite‐DS database [46], a multi‐binding‐site dataset derived from UniProt, and have identified multiple distinct binding sites on P00918. We then constructed test pairs by combining these sites with specific molecules. We input the pocket list together with the protein and ligand sequences to perform site‐specific affinity prediction. To ensure the reliability of the results, we additionally performed parallel protein‐ligand docking with AutoDock Vina for comparison. As shown in Figure 3, we present both the predicted affinity values and the Vina docking scores for each binding position. Given the dimensional disparity between Vina docking scores and MolDBG affinity predictions, we negated the Vina scores to align the correlation direction, thereby facilitating the assessment of MolDBG's capacity to discriminate binding strengths across different regions. The analysis yielded a CI value of 0.7124 and a Pearson of 0.6010, indicating that the model possesses a robust ability to distinguish affinity variations across distinct binding sites. Furthermore, we examined experimental results for the ligand VWW (ChemBank ID: 444051) across different binding regions of the UniProt target, providing a detailed visualization in Figure 3. We observed that MolDBG achieved consistent and accurate affinity predictions across most of the varying protein binding sites. Regarding the discrepancy observed between Site 2 and Site 20, we attribute this to minor variations between the binding sites predicted by MolDBG and the interacting residues identified during docking; we consider this deviation acceptable. Overall, these results demonstrate that our model achieves binding‐site‐level drug‐target affinity prediction, offering greater accuracy than existing target‐aware affinity prediction models.

### 2.4 Small Molecule‐Protein Interaction Binding Sites Prediction Performance

**FIGURE 4:**
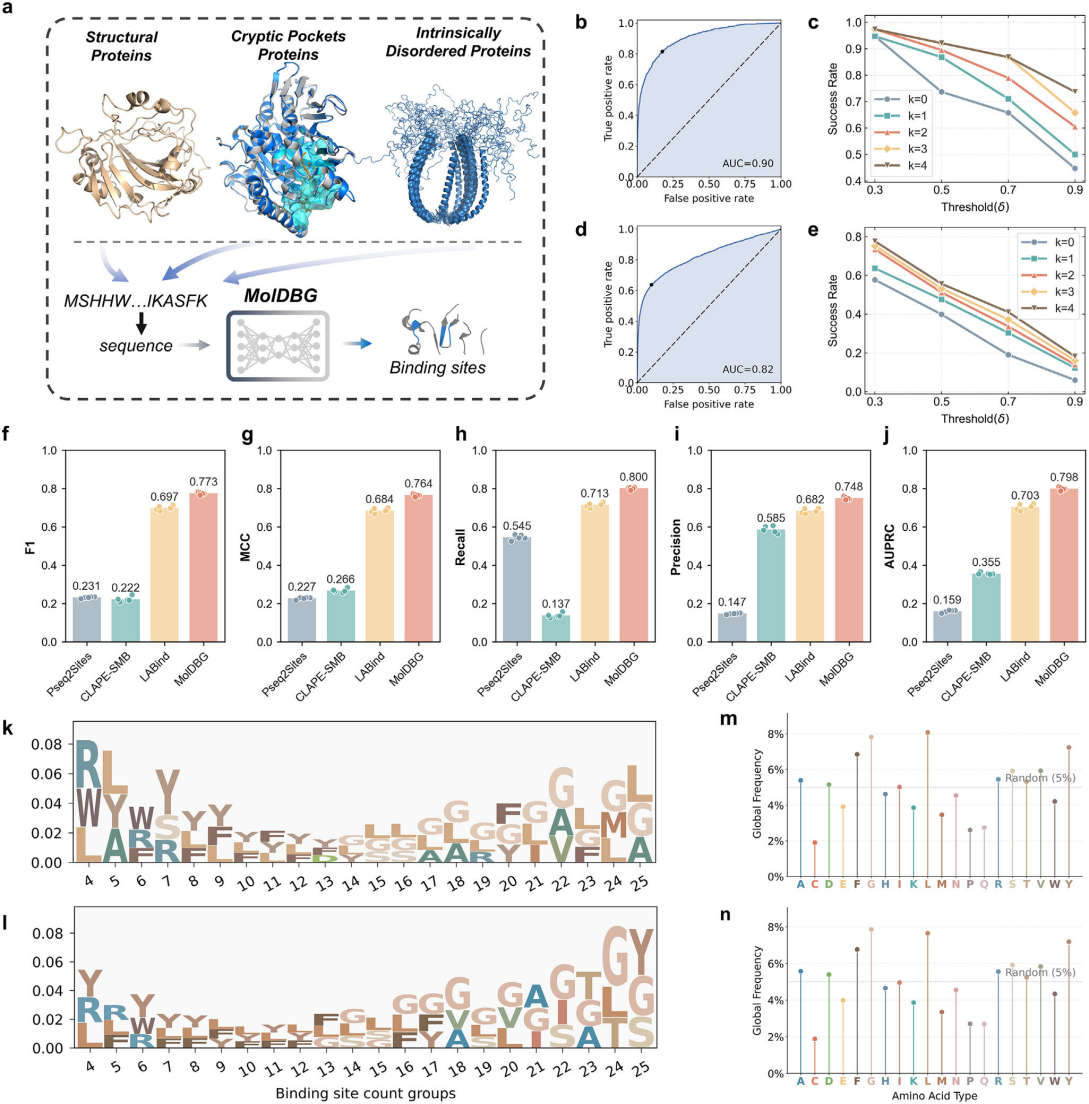
Performance evaluation of binding site prediction across diverse protein categories. (a) Binding site prediction pipelines for structural proteins, cryptic pocket proteins, and intrinsically disordered proteins. (b,d) AUROC curves for binding site prediction on the PocketMiner (b) and UniProtKB‐IDP (d) datasets. (c,e) Sample‐level success rates on the PocketMiner (c) and UniProtKB‐IDP (e) datasets across varying tolerance levels (k) and thresholds (δ). (f–j) Binding site prediction performance across five metrics, evaluated by 5‐fold cross‐validation on the BioLip‐BS dataset. The scatter points overlaid on each bar represent the results of individual folds, while the numerical values atop the bars indicate the mean performance. (k,l) Sequence logos of binding sites predicted by MolDBG (k) and derived from ground‐truth annotations (l). The x‐axis is the residue position within the binding‐site grouping and the y‐axis is information entropy (bits); amino acids use single‐letter codes, and only the three highest‐entropy residues per position are shown. (m,n) Distribution of binding‐site amino‐acid types predicted by MolDBG (m) and annotated in the BioLip‐BS dataset (n).

MolDBG architecture forces the model to learn representations of latent, interacting protein residues for binding site prediction, thereby providing reliability and interpretability for DTA prediction and molecule generation. We benchmarked MolDBG against three protein binding site prediction models: CLAPE‐SMB [47], Pseq2Sites [48], and LABind [21]. To ensure a rigorous comparison, we performed 5‐fold cross‐validation on all baseline models for the BioLip‐BS dataset, and the results are presented in Figure 4f–j. MolDBG demonstrated superior performance compared to existing methods across all metrics. Compared to LABind, the best‐performing concurrent baseline, MolDBG achieved substantial improvements of 12% in recall and 10% in precision for positive binding sites. This suggests that the auxiliary tasks of affinity prediction and targeted molecule generation synergistically improve the performance of binding site prediction, a finding consistent with previous studies that employed binding region prediction to enhance affinity accuracy [49]. Furthermore, leveraging the multi‐task framework and the design of the LSQM module, the model achieved an AUPRC of 0.7983 and an MCC of 0.7640, securing the highest scores across most positive sampling evaluation metrics. Conversely, Pseq2Sites and CLAPE‐SMB demonstrated relatively poor performance on the BioLip‐BS. We attribute this to their exclusive reliance on protein physicochemical properties and their failure to account for binding molecular information and multiple binding site scenarios. These results demonstrate that MolDBG, guided by implicit representations during pre‐training and explicit labels during fine‐tuning, is fully capable of capturing the representations of pockets or residue clusters where small molecule ligands actually bind. To enhance interpretability in molecule generation and affinity prediction, we provide simultaneous binding site prediction results as a reference during inference.

To assess the model's capacity to capture the physicochemical features of binding sites, we compared the sequence logo distributions of the ground‐truth binding sites (Figure 4l) with those predicted by MolDBG (Figure 4k). As shown in Figure 4k, the amino‐acid preference distribution predicted by our model is in high agreement with the ground‐truth labels in overall trend, successfully reproducing the sequence‐conservation features across different binding‐site count groups. Specifically, in the groups of lower binding site counts (Groups 4–10), the model acutely captured the dominance of aromatic amino acids with bulky side chains (e.g., Tyrosine [Y], Tryptophan [W]) and positively charged Arginine (R), aligning with the enrichment of Y, R, and L observed in Figure 4l. In contrast, in the groups of higher counts (Groups 20‐25), the model accurately recapitulated the significant enrichment of Glycine (G) and correctly predicted the distribution patterns of Tyrosine (Y) and Alanine (A). Furthermore, the model successfully fitted the high diversity and low conservation characteristics of the intermediate region (Groups 12‐18), as evidenced by the lower logo heights. This precise replication of amino acid distribution rules across the count groups provides compelling evidence that MolDBG not only achieves high prediction accuracy but also effectively learns the latent biochemical principles of the protein‐ligand binding interface, enabling it to infer reasonable residue types based on the local environment. Furthermore, the amino‐acid‐type distribution plots for the ground‐truth labels (Figure 4n) and the MolDBG predictions (Figure 4m) further corroborate that the model has successfully learned the distribution of amino‐acid categories that characterize genuine protein‐ligand interactions.

### 2.5 Cryptic Pocket Proteins Binding Site Prediction

We further evaluated the generalization capability and robustness of MolDBG at the binding sites of cryptic pocket proteins. We introduce the PocketMiner dataset [50], which comprises cryptic pockets formed through diverse conformational changes. Derived from the Protein Data Bank (PDB), this dataset identifies 38 apo‐holo protein structure pairs that exhibit significant root‐mean‐square deviation between protein states. This test set covers a diverse landscape of cryptic pocket structures and dynamics, serving as an effective benchmark for evaluating our model's performance on cryptic pocket proteins. We processed the sequences and pocket annotations for these 38 PDB entries. To explore potential cryptic pockets within the native protein while avoiding ligand‐induced bias, we primarily use binding site prediction outputs generated concurrently with the molecular design task. Notably, no ground‐truth ligand is used during inference; the pseudo‐ligand embedding is sampled solely from N(0,I) and processed by the DTALayerNorm with a given affinity value before driving the LSQM query. MolDBG demonstrated superior performance on the PocketMiner dataset, achieving a precision of 0.8351. Notably, as shown in Figure 4b, it outperformed the original PocketMiner model, attaining an AUROC of 0.9014 compared to 0.87. Furthermore, we introduced a Success Rate (SR) metric for sample‐level evaluation. We assessed each PDB structure individually (detailed results are provided in Table S5) and found that MolDBG achieved a sample‐level identification SR of 73.68% (with tolerance k=0 and threshold δ=0.5). We also visualized sample‐level SR across varying tolerance levels (k) and thresholds (δ), as shown in Figure 4c. These results demonstrate that MolDBG delivers robust binding‐site predictions for cryptic pockets, remaining resilient in the absence of explicit structural data or proteins with conformational dynamics. Consequently, the model could in principle be extended to affinity prediction and targeted molecule generation involving cryptic pocket proteins.

### 2.6 Disordered Protein Binding Sites Prediction

Drug‐target binding affinities or functional potencies are typically determined via methods such as Surface Plasmon Resonance or Enzyme Activity Assays. These techniques rely on observable signal changes derived directly from physicochemical interactions or functional readouts to quantify the actual binding strength, including experiments involving IDPs. However, IDPs lack stable three‐dimensional structures, rendering existing deep learning affinity prediction methods inapplicable. Consequently, we extended the MolDBG framework to generalize from proteins with stable conformations to the prediction of binding sites on disordered proteins. Following the prior study [47], we utilized an IDP dataset annotated from the UniProtKB‐IDP database, comprising 336 non‐redundant sequences with small‐molecule binding site annotations. Although most annotated sites in the UniProtKB‐IDP test set bind nucleotides, their derivatives, or coenzymes, and fewer than 15% involve clinical drug molecules, nucleotide [51] and coenzyme [52] binding pockets are nonetheless highly representative orthosteric sites that are widely exploited for small‐molecule therapeutics. Similar to the cryptic‐pocket binding‐site prediction experiment, we input the full protein sequences together with a given affinity value to simulate molecule generation for performing binding‐site prediction. As shown in Figure 4d, our model achieved an AUROC of 0.8187 at the binding‐site level. Notably, MolDBG successfully identified binding sites in nearly 200 samples (56.55%). Although the overall performance on the IDP dataset was modest compared with that on stable proteins, we attribute this primarily to the difference in binding patterns between clinical drug molecules and nucleotides, their derivatives, or coenzymes. Furthermore, acknowledging the variability and dynamic nature of ligand binding sites within the same receptor, we employed the SR metric for sample‐level evaluation, and the results are shown in Figure 4e. At the threshold (δ) of 0.3 with a tolerance of k=2, the model achieved sample‐level identification SR of 72.62%, demonstrating robust predictive capability for disordered protein binding. We believe that these results represent a successful generalization of the MolDBG architecture from structured protein‐ligand interactions to IDP‐small‐molecule scenarios, highlighting its potential to facilitate DTA prediction and molecule generation for IDPs.

### 2.7 Random Molecule Generation Performance

**FIGURE 5:**
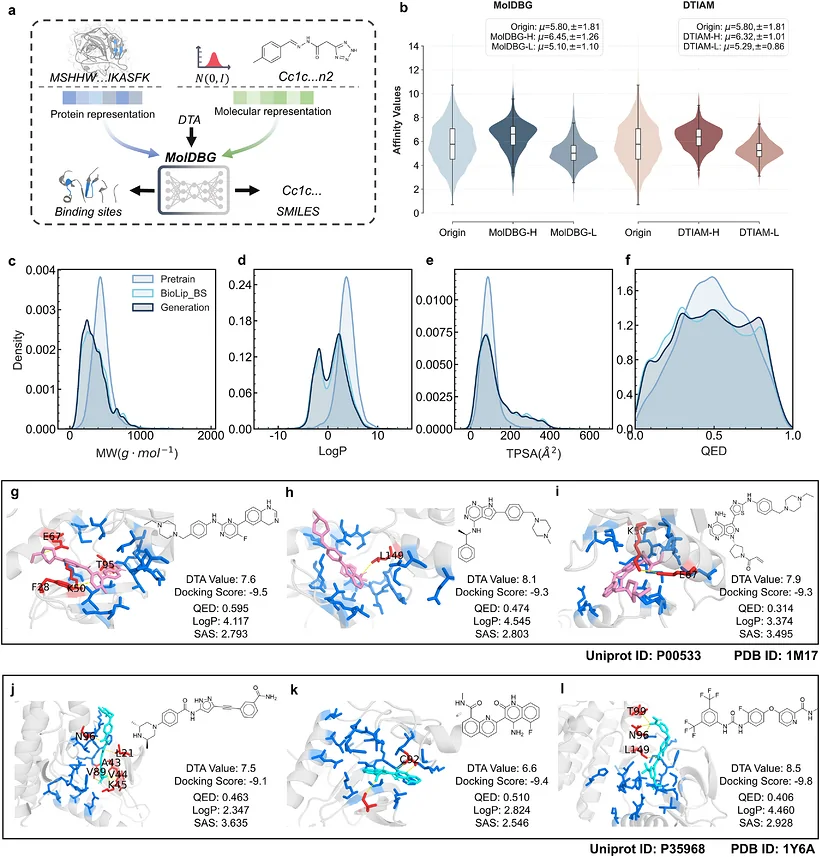
Evaluation of molecule generation and optimization capabilities. (a) The pipeline of molecule generation tasks, encompassing both lead optimization and de novo design. (b) Statistical comparison of predicted vs. ground truth affinities. Violin plots illustrate the distributions of affinities predicted by MolDBG (left) and DTIAM (right) across the two generated molecular datasets. (c–f) Physicochemical property distributions for the training sets vs. MolDBG‐generated molecules, including the Molecular Weight (MW), Wildman‐Crippen partition coefficient (LogP), Topological Polar Surface Area (TPSA) and Quantitative Estimate of Drug‐likeness (QED). The MolDBG set comprises 10 000 molecules generated from random hypotheses, while the comparison set consists of molecules randomly sampled from the two training sets. (g–l) Docking poses of conditionally generated molecules for EGFR (g–i) and VEGFR2 (j–l). Residues in **red** denote interaction sites from AutoDock Vina docking; residues in **blue** denote binding sites predicted by MolDBG.

For the molecule generation task, we benchmark our framework against several representative generative models. To evaluate MolDBG molecule generation performance, we randomly selected target proteins from the held‐out validation fold and sampled random affinity scores to construct pseudo‐ligand representations, generating a total of 10 000 molecules. We also included the MolDBG‐Vanilla to assess the impact of pre‐training on the performance of the molecular design module across large‐scale paired datasets. Evaluation metrics are calculated according to the protocols established by PGMG [5] to ensure a fair comparison. DeepDTAGen is excluded from the table because the limited size of the training set prevented the model from effectively converging on the generation task. The results are presented in Table 1, and reveal that MolDBG achieved performance comparable to PGMG [5] and REINVENT 2.0 [53] across multiple metrics. Moreover, compared to SyntaLinker [54], Pocket2Mol [33], DEVELOP [55], and LS‐MolGen [56], our model demonstrated a substantial improvement in the Ratio of Available Molecules (RAM). Notably, Pocket2Mol and DEVELOP exhibited lower Uniqueness scores. We attribute this to their pocket‐based generation strategies, where the fixed sampling space of a given protein structure biases the design toward a specific class of molecules that strictly conform to the pocket geometry. The inclusion of the MolDBG‐Vanilla baseline further validates the efficacy of our staged training strategy. The results indicate that the extensive chemical space in BindingDB substantially enhanced the generation module's decoding capabilities, resulting in an approximately 6.6% improvement in Validity for MolDBG compared to the vanilla version. Compared to existing generative models, MolDBG surpassed PGMG by approximately 2.5% in validity. While the Novelty scores were modest, we attribute this primarily to minor overfitting introduced by data augmentation during fine‐tuning. Additionally, the stochastic generation process in MolDBG is inherently constrained by the specific target sequences and affinity conditions, thereby naturally limiting deviations from the known distribution. Nevertheless, these results confirm the model's ability to generate valid drug candidates. Furthermore, we performed a statistical analysis of four key physicochemical properties comparing the generated molecules against the two training datasets (BindingDB and BioLip‐BS): the Molecular Weight (MW), Wildman‐Crippen partition coefficient (LogP), Topological Polar Surface Area (TPSA), and Quantitative Estimate of Drug‐likeness (QED). As illustrated in Figure 5c–f, MolDBG effectively reproduced the physicochemical distributions of the BioLip‐BS dataset.

**TABLE 1:** Performance comparison of molecule generation tasks.

Model	Validity	Uniqueness	Novelty	RAM
SyntaLinker [54]	**100.0%**	88.0%	90.3%	79.5%
Pocket2Mol [33]	**100.0%**	34.9%	99.7%	34.8%
DEVELOP [55]	85.6%	33.4%	99.7%	28.6%
LS‐MolGen [56]	63.2%	**100.0%**	76.1%	48.1%
REINVENT 2.0 [53]	96.3%	99.9%	99.9%	**96.1%**
PGMG [5]	94.8%	99.9%	**100.0%**	94.8%
**MolDBG‐Vanilla**	90.7%	95.6%	88.2%	76.5%
**MolDBG**	97.3%	98.1%	91.1%	87.0%

*Note*: For molecule generation, the model utilized protein sequences from the held‐out validation fold and randomly sampled affinity values. Bold values indicate the best performance in each column. The proposed models are highlighted in bold.

### 2.8 Affinity‐Based Conditional Molecular Design

We also evaluate the performance of molecule generation conditioned on specific affinity values and targets. By using random molecular seeds and target proteins from the held‐out validation fold, we aimed to design molecules capable of achieving specified binding strengths with their targets, theoretically enabling stable coverage of all test target proteins through repeated generation. In practical pharmaceutical development, design efforts typically focus on two distinct scenarios: high‐affinity (targeting) and low‐affinity (non‐targeting) design. Accordingly, we defined high‐affinity and low‐affinity thresholds based on the 7.1 (75^th^ percentile) and 4.5 (25^th^ percentile) affinity scores from the original dataset, respectively. Using these thresholds, we generated two molecular libraries comprising 10 000 small molecules each: HDTAM (High DTA‐based generated Molecules) and LDTAM (Low DTA‐based generated Molecules). To rigorously assess whether the actual drug‐target affinities of the generated molecules aligned with the conditional inputs, we employed the DTIAM affinity prediction model as an external validator. We predicted the affinities for both HDTAM and LDTAM samples using MolDBG and DTIAM, and subsequently performed a statistical analysis comparing these predictions with the distribution of the original test dataset. As illustrated in Figure 5b, for the conditionally generated HDTAM sets, the overall means of affinities predicted by MolDBG and DTIAM were 6.45 and 6.32, respectively. Notably, MolDBG exhibited a tighter distribution around the high and low quartiles. Furthermore, the affinities predicted by MolDBG for the HDTAM and LDTAM molecules clustered more closely around the specified high‐affinity and low‐affinity thresholds, respectively. These results demonstrate that the model possesses robust conditional affinity design capabilities and reaffirm the stability of its affinity prediction module, thereby offering a viable pathway for high‐affinity optimization based on molecular seeds.

Furthermore, to evaluate the capacity of the model for conditional molecule generation, we used specific affinity values and target protein sequences as inputs to conduct an affinity‐based molecular generation case study. We selected VEGFR2 (UniProt ID: P35968, PDB ID: 1Y6A) and EGFR (UniProt ID: P00533, PDB ID: 1M17) as representative targets to validate the conditional design capability of MolDBG. To ensure a rigorous assessment, AutoDock Vina was employed as an independent evaluator of binding affinity. From the generated molecules, we select six novel candidates (the top three per target) that are absent from both the pre‐training and fine‐tuning datasets, and then rank them by QED value. The top three candidates for EGFR and VEGFR2 are presented in Figure 5g–i and Figure 5j–l, respectively. It should be noted that while higher conditional affinity values indicate stronger binding, lower Vina scores correspond to higher binding stability. Meanwhile, MolDBG explicitly models drug‐target interactions and predicts binding sites concomitantly with molecule generation. To validate this, we visualized the Vina‐calculated docking poses relative to the target proteins and compared the computed interaction residues with those predicted by MolDBG. As illustrated in the figures, the physical interaction sites determined by Vina docking exhibit a high degree of spatial overlap with the binding sites predicted by our model. These results demonstrate that MolDBG effectively generates drug candidates with specified binding affinities for given protein sequences, and offers interpretable interaction insights for these molecules. In addition, we provide a detailed structural‐novelty analysis of the generated molecules in Section S7, including a 2D structural comparison against the closest training‐set analog, to demonstrate that our model produces genuinely novel designs.

## 3 Discussion

While deep learning has accelerated both drug‐target affinity and de novo design of high‐affinity drugs, target‐aware representations are insufficient for the precise physicochemical interactions required for residue‐level binding. MolDBG explicitly couples molecular design and affinity screening with binding‐site identification based on a unified multi‐scenario framework. Similar to traditional medicinal chemists' drug design processes, MolDBG also demonstrates that site‐specific constraints are not merely supplementary features but fundamental prerequisites for robust molecular generation and affinity prediction. MolDBG can not only predict affinity but also dynamically distinguish binding strengths across distinct pockets for the same ligand. This extra site‐level predictive resolution provides an important interpretability benefit, allowing researchers to verify where the model posits the interaction occurs. While MolDBG shows clear advantages, several limitations remain. First, due to the inherent constraints imposed by strictly matching specific target sequences and affinity values during fine‐tuning, the Novelty scores of the generated molecules suggest a slight tendency toward conservatism. Future iterations of generative networks aim to encourage broader exploration of uncharted chemical space while maintaining high affinity. Second, although the sequence‐based approach performs well on structured proteins and extends to IDPs and cryptic pockets, it inherently lacks the explicit spatial geometry (e.g., precise atomic distances and bond angles) necessary for late‐stage lead optimization. The lightweight, dynamic 3D‐conformational sampling modules are also important for enabling direct docking‐based validation. Furthermore, the development of de novo design methods and evaluation benchmarks specifically tailored to IDPs and cryptic pockets remains a priority. In the future, multi‐task deep learning frameworks that incorporate molecular understanding and design tasks will continue to accelerate the discovery of therapeutic agents as single‐modality PLMs evolve and high‐quality wet‐lab experimental data expand. In addition, ensemble docking, homology modeling, and conformational sampling are also promising directions for structure‐aware validation of dynamic targets in future work.

## 4 Experimental Section

### 4.1 Datasets

Our primary datasets stem from the BindingDB and Q‐BioLiP [57] public databases. For the pre‐training phase, we utilized BindingDB, a standard drug‐target affinity dataset containing experimentally determined binding data. We set the maximum sequence lengths for molecules and proteins to 256 and 1024 tokens, respectively. After removing redundant sequences and invalid affinity entries, the pre‐training dataset comprised over 1.4 million drug‐target pairs after filtering. Notably, $IC_{50}$ values served as the primary metric of affinity during this phase. For the fine‐tuning phase, we constructed a dataset, BioLip‐BS, based on Q‐BioLiP, a database of ligand‐protein interactions derived from quaternary structure. The Q‐BioLiP database includes protein‐small‐molecule complexes with experimentally measured or docked affinity values (e.g., $K_{i}$, $K_{d}$, or $IC_{50}$). By performing rigorous structure, sequence, and binding‐site alignment checks on Q‐BioLiP, we filtered out entries with verified binding‐site annotations and valid affinity values. Each sample has four data points: protein, SMILES, affinity, and pocket index. We treat it as a new sample if any data point differs; this finally yielded a specialized 11 045‐sample dataset, BioLip‐BS. The detailed dataset statistics are summarized in Table S1 and Figure S1. All affinity metrics were converted to their negative logarithmic forms (e.g., $pK_{d}$). To resolve dimensional discrepancies between different affinity metrics, we applied Z‐score normalization. To mitigate the limited size of the fine‐tuning dataset, we employed a randomized SMILES augmentation strategy. By perturbing the atomic traversal order to generate diverse representations of identical molecules, we expanded the dataset by 10‐fold, yielding approximately 110 000 high‐quality training samples. Furthermore, to evaluate affinity variations for identical protein‐ligand pairs across distinct binding sites, we utilized the Unique Protein‐centric ligand binding site dataset from UniSite‐DS [46] for multi‐site annotations. Structural data for Carbonic Anhydrase II (CA2; UniProt ID: P00918), selected for our case study, were retrieved directly from UniSite‐DS. The structures of the targets VEGFR2 (PDB ID: 1Y6A) and EGFR (PDB ID: 1M17) were downloaded from the PDB database.

### 4.2 Model Architecture

#### 4.2.1 Representation of Proteins and Molecules

To capture the precise physicochemical features of protein‐ligand interactions, we employed distinct pre‐trained models to extract residue‐ and token‐level representations. Specifically, the pre‐trained ESM‐2 (Evolutionary Scale Modeling 2) model served as the backbone protein encoder. We froze its parameters to extract contextual representations of amino acid sequences. ESM‐2 was trained on the UniRef50 dataset, which comprises over 60 million protein sequences covering structurally stable proteins, IDPs, and those containing cryptic pockets. This extensive pre‐training equips MolDBG with robust representational capabilities, enabling generalization across diverse and challenging protein types. For molecular representation, we utilized the MolFormer pre‐trained model to extract molecular embeddings. Given a protein sequence $P=(p_{1},p_{2},\text{…},p_{L_{p}})$ and a molecular SMILES string $M=(m_{1},m_{2},\text{…},m_{L_{m}})$, the encoders generate residue‐ and token‐level embeddings. Let $P_{prot}\in R^{L_{p}\times d_{p}}$ and $M_{mol}\in R^{L_{m}\times d_{m}}$ denote the residue‐level protein embedding produced by ESM‐2 and the token‐level molecular embedding produced by MolFormer, respectively, where $L_{p}$ is the protein sequence length (maximum 1024). They are defined as:

(1) $$\begin{array}{cc} & P_{\mathrm{prot}}=\text{ESM2}\left(P\right)\in R^{L_{p}\times d_{p}}\\ & M_{\mathrm{mol}}=\text{MolFormer}\left(M\right)\in R^{L_{m}\times d_{m}} \end{array}$$

where $d_{p}=768$ and $d_{m}=512$ denote the embedding dimensions for protein residues and molecular tokens, respectively. $L_{p}=1024$ and $L_{m}=256$ represent the maximum sequence lengths for proteins and molecular SMILES strings. To unify the feature dimensions across modalities, we applied a linear projection layer to map both protein and molecular features into a shared embedding space $R^{d}$ (d=768). This process yields token‐wise representations suitable for subsequent cross‐modal interaction.

#### 4.2.2 Drug‐Target Interaction Learning Module

The conditional affinity molecular design task aims to generate targeted molecules with specified binding strengths based on target proteins and affinity data, whereas the affinity prediction task estimates binding strength using protein sequences and small molecule SMILES. Fundamentally, both tasks hinge on elucidating the molecular mechanisms governing ligand binding at specific protein sites. To unify these objectives, we developed a framework that integrates a binding site prediction task to provide explicit gradient guidance, compelling the intermediate module to focus on feature extraction within local drug‐target interaction regions. First, to model the selective binding of small molecules to potential sites on the protein surface, we developed an attention‐based LSQM module. This module utilizes the molecular embedding $M_{mol}$ (representing either a real or pseudo‐ligand) as the query vector, while the residue‐wise protein embedding $P_{prot}$ serves as both Key and Value. Through a cross‐attention mechanism, the LSQM extracts representations of potential interacting residues:

(2) $$\begin{array}{c} Q=W_{Q}M_{mol},\;K=W_{K}P_{prot},\;V=W_{V}P_{prot}\\ H_{query}=\text{softmax}\left(\frac{QK^{T}}{\sqrt{d}}\right)V \end{array}$$

Here, $H_{query}\in R^{L_{m}\times d}$ denotes the contextual latent binding features within the protein‐binding scenario, capturing the compatibility of molecular fragments at the binding‐site level.

For the conditional molecular design task, we generate a pseudo‐ligand embedding, $M_{sample}$, via random sampling. To incorporate conditional information, we perturb $M_{sample}$ using the target affinity value through a DTA‐based Conditional LayerNorm (DTALayerNorm) module. This process achieves the preliminary injection of conditional information, yielding the conditioned representation ${\tilde{M}}_{mol}$:

(3) $$\begin{array}{c} \gamma \left(c\right)=W_{\gamma ,2}\;\phi \left(W_{\gamma ,1}\;c+b_{\gamma ,1}\right)+b_{\gamma ,2}\\ \beta \left(c\right)=W_{\beta ,2}\;\phi \left(W_{\beta ,1}\;c+b_{\beta ,1}\right)+b_{\beta ,2}\\ \text{DTALayerNorm}\left(x;c\right)=\gamma \left(c\right)⊙\frac{x-\mu }{\sigma }+\beta \left(c\right) \end{array}$$

where c∈R is the scalar conditional signal constructed from the given affinity value; γ(·) and β(·) are two‐layer MLPs that map the affinity condition to channel‐wise scale and shift parameters, with $W_{\gamma ,1},W_{\beta ,1}\in R^{D\times 1}$ and $W_{\gamma ,2},W_{\beta ,2}\in R^{d\times D}$ as learnable weights and $b_{\gamma ,\cdot },b_{\beta ,\cdot }$ as bias terms; D is the hidden dimension of the conditional MLP (set to d/6); ϕ denotes the GELU activation; $\frac{x-\mu }{\sigma }$ is a LayerNorm without learnable affine parameters (with μ and σ the mean and standard deviation of x over the feature dimension); and ⊙ denotes element‐wise multiplication.

We employ the concatenated vector $M_{batch}=\left[M_{mol},{\tilde{M}}_{mol}\right]$ as the Query to construct latent binding residues for both real and pseudo‐ligands. Notably, during training for the specified binding site task, we impose a masking constraint on the protein representation, preventing the LSQM from attending to tokens outside the designated binding pocket. Unlike single‐task or alternating optimization approaches, MolDBG jointly models affinity prediction and conditional design. This strategy enables the fusion layer, guided by multi‐task objectives, to deeply mine interaction details at the residue level. To effectively integrate features from distinct pre‐trained models, we adopted the information injection strategy from [58], incorporating rotary positional encodings and modality‐specific encodings into both the latent protein binding representation $H_{query}$ and the molecular representation. Finally, we designed a mixed transformer encoder to extract latent drug‐target binding features, yielding the joint semantic representation H:

(4) $$\begin{array}{c} H=\text{concat}\left[\;H_{query},\;M_{q}\;\right],\;M_{q}\in \left\{M_{mol},\;{\tilde{M}}_{mol}\right\}\\ H_{fusion}=\text{Mix-Encoder}\left(H+X_{i}^{modal}+E_{pos}\right),\;i\in \left\{0,1\right\} \end{array}$$

where $M_{q}$ denotes the molecular embedding used as the LSQM query; $H_{query}$ is the corresponding LSQM output, and concatenating it with $M_{q}$ along the sequence dimension yields the length‐$2L_{m}$ fused input. Mix-Encoder(·) denotes the multi‐layer mixed Transformer encoder that performs cross‐modal self‐attention over the concatenated latent‐binding and molecular tokens, $E_{pos}\in R^{2L_{m}\times d}$ represents the rotary positional encoding, and $X_{i}^{modal}\in R^{2\times d}$ denotes learnable modality encodings used to distinguish between molecular tokens and amino acid residue tokens.

#### 4.2.3 Multiple Decoder Design

Building on the drug‐target interaction representations, we constructed three distinct task decoders for drug affinity prediction, binding site prediction, and conditional molecule generation. First, for the Molecular Decoder (Mol‐Decoder), we employed an autoregressive Transformer architecture to reconstruct molecular SMILES sequences from the affinity‐perturbed joint binding features. Acknowledging that conditional affinity information might degrade due to the substantial depth of the Query and fusion modules, we re‐injected affinity information into the fusion module's output features via the DTALayerNorm. The Mol‐Decoder generates molecules by predicting the conditional probability of the next token:

(5) $$p\left(M|{\tilde{H}}_{fusion}\right)=\prod_{t}p\left(m_{t}|m_{<t},{\tilde{H}}_{fusion}\right)$$

where ${\tilde{H}}_{fusion}$ denotes the conditioned representation derived from the interaction joint representation $H_{fusion}$ following affinity injection.

Second, we designed a Binding Sites Decoder (BS‐Decoder) to predict the actual molecular binding sites within the protein structure. To ensure explicit gradient guidance from the binding site prediction task to the LSQM module, the BS‐Decoder module is built directly on the LSQM outputs. Internally, the BS‐Decoder utilizes a lightweight cross‐attention mechanism to project the $H_{query}$ representations back into the protein sequence dimension. Dimensionality reduction is achieved via simple 1D convolution and dense neural networks, thereby avoiding the information loss associated with direct weighting or mean pooling. The module ultimately outputs the probability of each protein residue binding to the input molecule.

Finally, for the affinity prediction task, we developed the Drug‐Target Affinity Mixture of Experts Decoder (DTA‐Decoder). By incorporating an MoE architecture, this module enables multiple experts to model heterogeneous and complex protein‐ligand interaction patterns, thereby enhancing prediction performance and cold‐start generalization. The module also operates on the interaction representation vector $H_{fusion}$ from the drug‐target interaction module. First, the gating network generates a routing distribution across multiple experts based on the interaction vector $H_{pool}$, which is derived from $H_{fusion}$ via attention pooling:

(6) $$g=\text{softmax}(W_{g}\cdot x)$$

where $W_{g}\in R^{N_{e}\times d}$, $N_{e}$ is the number of experts, and x corresponds to the input $H_{pool}$. Given that interactions involve heterogeneous and mixed binding modes, we employ a Top‐k strategy to activate experts for guided binding strength prediction. Each expert consists of a two‐layer MLP:

(7) $$y_{i}=f_{i}\left(x\right)=W_{i,2}\sigma \left(W_{i,1}x\right)$$

The final output representation is given by:

(8) $$\hat{y}=\text{DenseLayer}\left(\sum_{i\in \text{Top-}k}\alpha_{i}y_{i}\right),\;\alpha_{i}=\frac{e^{g_{i}}}{\sum_{j\in \text{Top-}k}e^{g_{j}}}$$

where $W_{i,1}\in R^{d\times d}$ and $W_{i,2}\in R^{d\times d}$ are learnable parameters within the i‐th expert, σ is the activation function, $y_{i}$ is the expert output, and $\alpha_{i}$ is the gating probability. The DenseLayer is a simple non‐linear network constructed from multiple MLP layers. Ultimately, the affinity value is non‐linearly fit using a DenseLayer based on the aggregated expert outputs. By introducing the MoE architecture, the model adaptively activates distinct sub‐networks to accommodate complex binding modes, consistently improving sensitivity in high‐affinity regions.

### 4.3 Multi‐Task Learning and Loss Functions

The model employs a multi‐task joint training strategy to foster knowledge sharing and representational synergy across distinct subtasks. The overarching objective is to minimize the aggregate loss derived from drug‐target affinity prediction, binding site identification, and conditional molecular design. The total objective function is formulated as follows:

(9) $$L=L_{Gen}+L_{BS}+L_{DTA}$$

where $L_{Gen}$, $L_{BS}$, and $L_{DTA}$ denote the loss components for molecule generation, binding site prediction, and affinity prediction, respectively.

Following established protocols for drug‐target affinity prediction, we employ MSE loss as the optimization objective to minimize the deviation between predicted and experimentally measured affinities. To align the magnitude of $L_{DTA}$ with the losses of the other tasks, we applied Z‐score standardization to both the affinity predictions and their corresponding labels.

(10) $$L_{DTA}=\frac{1}{N}\sum_{i=1}^{N}{\left(\varphi \left(y_{i}\right)-{\hat{y}}_{i}\right)}^{2}+\lambda \cdot L_{MOE\_balance}$$

where $y_{i}$ and ${\hat{y}}_{i}$ denote the ground‐truth affinity value and the model prediction, respectively, and φ(·) is the Z‐score standardization function; the affinity head directly outputs predictions in the standardized space. $L_{MOE\_balance}$ is the auxiliary load‐balancing loss defined as the squared sum of expert gating probabilities, ensuring uniform utilization of all experts [59], weighted by the coefficient λ.

Similarly, for molecule generation, we utilized the standard cross‐entropy loss for optimization:

(11) $$L_{Gen}=-\frac{1}{T}\sum_{t=1}^{T}p_{t}\log q\left(m_{t}|m_{1:t-1},c\right)$$

where p represents the probability distribution of the ground truth tokens, and q denotes the probability distribution generated by the Mol‐Decoder for each token $m_{t}$, conditioned on the preceding tokens $m_{1:t-1}$ and the affinity condition c.

Regarding binding site prediction, assuming independence among positions on the protein sequence, we formulated the task as a residue‐wise binary classification problem, employing cross‐entropy loss to guide the learning of actual binding sites. Given that binding sites typically constitute about 4%–5% of total protein residues, resulting in severe class imbalance, we pre‐compute positive sample weights based on the number of binding sites. Furthermore, recognizing that binding residues and non‐binding regions typically exhibit contiguous clustering rather than random distribution, we incorporated the Dice Loss [60], widely used in image segmentation, to effectively capture the structural differentiation between binding and non‐binding regions.

(12) $$\begin{array}{c} \begin{array}{c} L_{BS}=-\frac{1}{N}\sum_{i=1}^{N}w_{i}\left[y_{i}\log{\hat{y}}_{i}+\left(1-y_{i}\right)\log\left(1-{\hat{y}}_{i}\right)\right]\\ +\left(1-\frac{2\sum_{i}y_{i}{\hat{y}}_{i}+\text{smooth}}{\sum_{i}\left(y_{i}+{\hat{y}}_{i}\right)+\text{smooth}}\right) \end{array} \end{array}$$

where $w_{i}$ denotes the positive or negative sample weight derived from the binding site ratio; ${\hat{y}}_{i}$ and $y_{i}$ represent the predicted probability (output by the BS‐Decoder) and ground truth label for the i‐th residue being a binding site, respectively; and smooth is a smoothing factor. The first term of $L_{BS}$ corresponds to the token‐wise Binary cross‐entropy loss on the protein sequence, while the second term represents the Dice Loss, facilitating region‐level segmentation and identification across the full protein.

### 4.4 Training Details and Parameter Settings

For the dataset split, during the pre‐training stage we randomly partitioned the processed dataset into training and validation sets at a 9:1 ratio, and the checkpoint with the lowest affinity‐prediction RMSE on the validation set was retained for the second‐stage fine‐tuning. For the fine‐tuning stage, we randomly partitioned the BioLip‐BS dataset into training and validation sets at a 4:1 ratio to perform 5‐fold cross‐validation. Given the extreme scarcity of open‐source datasets that contain paired drug‐target affinity and binding‐site annotations, and the inherent difficulty of training an effective molecular generator on limited data, we adopted a two‐stage training strategy of pre‐training and fine‐tuning. During the pre‐training phase, we used protein‐ligand affinity data from BindingDB. The pre‐trained model was guided solely by the affinity‐prediction and conditional molecular‐design tasks, enabling it to learn drug‐target binding interaction constraints and molecular‐design capabilities. To enhance robustness, we employed a masking technique in which approximately 15% of the tokens in each protein input sequence were randomly replaced with the (MASK) token. For the generation task, we applied teacher forcing, in which ground‐truth tokens are used to predict the subsequent token. For the affinity‐prediction task, the model was trained with a regression head to predict scalar affinity values. The pre‐training objective combined an MSE loss for affinity and a cross‐entropy loss for molecular reconstruction. In the fine‐tuning stage, we introduced the Binding Sites Decoder to explicitly guide the model, concentrating the extraction of latent binding‐site representations within the LSQM module. By backpropagating binding‐site gradients into the LSQM, we optimized the model parameters for both the downstream affinity prediction and molecule generation tasks within the drug‐target interaction module. The entire model was optimized using the joint loss of all three tasks. We also describe the two‐stage pre‐training and fine‐tuning paradigm in Section S2. The training framework was implemented using PyTorch Lightning. We employed the Adam optimizer [61], utilizing distinct cosine decay learning rate schedules with warm‐up strategies for each stage to ensure smooth transitions between learning stages. Gradient clipping was applied with a maximum norm of 1.0. During pre‐training, the batch size was set to 128, with Adam parameters $\beta_{1}=0.9$ and $\beta_{2}=0.98$. The learning rate was linearly warmed up over the first 10 000 steps from $1\times 10^{-6}$ to a peak of $1\times 10^{-4}$, and then decayed to a minimum of $1\times 10^{-5}$ following the cosine schedule. Training spanned 15 epochs (approximately 160k steps) and required five days on eight NVIDIA V100 GPUs. Upon convergence of the first stage, the trained parameters were initialized for the second stage. For fine‐tuning, the batch size was set to 64, and Adam parameters were adjusted to $\beta_{1}=0.9$ and $\beta_{2}=0.999$. The learning rate was warmed up over the first 1500 steps from $1\times 10^{-6}$ to a peak of $1\times 10^{-4}$, and subsequently annealed to a minimum of $1\times 10^{-6}$ under the same cosine schedule. This phase also ran for 20 epochs (approximately 27k steps), with the optimal model selected based on the RMSE metric for affinity prediction. The overall model architecture remained consistent across both pre‐training and fine‐tuning stages. Detailed module hyperparameters and the LSQM hyperparameter ablation are provided in Section S3 (Tables S2 and S3) and Section S8 (Tables S7– S10), respectively.

### 4.5 Evaluation Metrics

To evaluate the binding site prediction task, following the prior study [21], we employed standard metrics including Accuracy (Acc), Recall (Rec), Precision, and the F1‐score (F1). Except for the Area Under the Receiver Operating Characteristic Curve (AUROC) and the Area Under the Precision‐Recall Curve (AUPRC), all other metrics require a specific threshold to binarize the predicted binding probabilities. This threshold was determined by maximizing the Matthews Correlation Coefficient (MCC), a metric we incorporated to ensure robust evaluation given the scarcity of positive samples. For the molecule generation task, we adopted metrics widely established in prior studies [5] to assess performance: Novelty, Uniqueness, Validity, and the Ratio of Available Molecules (RAM). Validity represents the percentage of generated SMILES strings that correspond to chemically valid molecules. Uniqueness measures the proportion of non‐repetitive valid molecules. Novelty denotes the percentage of chemically valid molecules absent from the training set. Finally, RAM is defined as the proportion of novel molecules relative to the total number of generated outputs. To assess affinity prediction performance, we used six metrics employed by a prior study [36]: Mean Squared Error (MSE), Root Mean Squared Error (RMSE), Concordance Index (CI), Pearson correlation coefficient (Pearson), Spearman's rank correlation coefficient (Spearman), and the modified squared correlation coefficient ($r_{m}^{2}$) metric. Furthermore, regarding independent test sets for cryptic pocket proteins and IDPs, annotation labels are typically defined at the pocket or regional level. In contrast, MolDBG is designed to identify precise residue‐level interaction sites rather than broad binding pockets. This distinction in granularity results in an inherent disparity in residue counts compared to ground‐truth binding site labels. To address this, we formulated a precision‐based Success Rate (SR) metric to evaluate performance at the sample level, and the definitions and calculation methods are presented in Section S4. In addition, our docking‐based validation relies on single static crystal conformations and is therefore appropriate only for structurally stable targets. For dynamic targets such as IDPs, we instead rely on the sequence‐based annotations of the datasets to validate binding‐site agreement.

### 4.6 Baseline Models

To rigorously evaluate the proposed architecture, we conducted extensive comparative experiments against representative baselines. For the molecule generation task, we benchmarked MolDBG against six representative models: SyntaLinker [54], Pocket2Mol [33], DEVELOP [55], LS‐MolGen [56], REINVENT 2.0 [53], and PGMG [5]. Specifically, SyntaLinker employs a Transformer architecture to generate novel compounds by learning structural information from paired molecular fragments. Pocket2Mol facilitates efficient molecular sampling by capturing the chemical bond relationships and space within the target binding pocket. DEVELOP integrates GNNs with CNN to embed 3D pharmacophoric constraints into the generation process, supporting both linker design and scaffold elaboration. LS‐MolGen leverages prior knowledge of bioactive compounds and target protein structures, employing a combination of representation learning, transfer learning, and reinforcement learning submodules for de novo drug design. REINVENT 2.0 introduces a reinforcement learning framework for constrained molecule generation, enabling the efficient exploration and exploitation of chemical space. Finally, PGMG incorporates pharmacophore guidance into molecular representations to facilitate the generation of bioactive molecules, demonstrating its applicability in both structure‐based and ligand‐based design paradigms. For the affinity prediction and binding site prediction tasks, we compared our approach against representative domain‐specific models. To ensure a fair and rigorous assessment, all baseline models were retrained and evaluated using 5‐fold cross‐validation on our dataset. In the field of drug‐target affinity prediction, MolDBG was compared against six representative methods: DeepDTA [12], GraphDTA [15], WGNN‐DTA [45], AttentionDTA [14], DTIAM [11], and DeepDTAGen [36]. DeepDTA employs a dual‐channel CNN to extract features from sequences for affinity prediction. With the advent of geometric deep learning, GraphDTA advanced this approach by modeling molecules as graphs. WGNN‐DTA further extended this by modeling both molecules and proteins as graph structures, enabling precise prediction via a graph network. AttentionDTA uses multi‐head attention to dynamically model interaction weights between drug and protein subsequences, thereby enhancing both accuracy and interpretability. DTIAM is a recent, comprehensive framework that employs a two‐stage pre‐training and fine‐tuning strategy that integrates drug‐target interaction, affinity prediction, and bioactivity classification, demonstrating superior performance in cold‐start scenarios. Finally, DeepDTAGen shares a conceptual similarity with our work by employing a multi‐task framework to simultaneously predict affinity and generate target‐aware drug variants. It utilizes a Variational Autoencoder architecture with gated convolutions and molecular graph networks to integrate these tasks. We also benchmarked against three recent representative binding site prediction models, including Pseq2Sites [48], CLAPE‐SMB [47], and LABind [21]. Similar to our approach, LABind integrates pre‐trained protein embeddings and molecular SMILES, utilizing a Graph Transformer to capture binding modes within the local protein context and cross‐attention mechanisms to learn ligand‐specific features. CLAPE‐SMB combines the ESM‐2 protein language model with Triplet Center Loss contrastive learning, achieving high‐precision prediction based solely on amino acid sequences. Pseq2Sites is a sequence‐only model that employs deep CNN to extract local features and position‐based attention mechanisms to capture long‐range dependencies between binding residues. Furthermore, to validate the efficacy of our two‐stage training strategy, we included the MolDBG‐Vanilla model as a baseline for experiments.

## Author Contributions

M.L. supervised the research. G.L. and M.L. conceived the initial idea. G.L. and C.W. collected and preprocessed the data. G.L. designed and trained the model. G.L. conducted the computational experiments and analyzed the results. G.L. and Q.Z. prepared the manuscript. G.L., Q.Z., C.W., Z.Y., A.J.W., J.T., and M.L. revised the manuscript. All authors read and approved the final manuscript.

## Conflicts of Interest

The authors declare no conflicts of interest.

## Supporting information


**Supporting File**: advs77238‐sup‐0001‐SuppMat.pdf.

## Data Availability

All source code of MolDBG and used data can be obtained from https://github.com/CSUBioGroup/MolDBG.
