# Supplementary material for: A Site‐Aware Representation Learning Framework For Unified Molecular Interaction Modeling and Generative Design
Source: Adv Sci (Weinh). 2026 Sep 10:e77238. Online ahead of print. doi: 10.1002/advs.77238 (PMC13562789; doi:10.1002/advs.77238)
Supplement: Supplementary file 1 [file ADVS-9999-e77238-s001.pdf]

Supporting Information:

A site-aware representation learning framework for unified molecular interaction modeling and generative design

Gang Luo[1], Qianqian Zhang[1], Chenhao Wang[1], Zhiheng Yi[1,2], Alex Jinpeng Wang[1], Jing Tang[3], and Min Li[1,*]

[1]*School of Computer Science and Engineering, Central South University, Changsha 410083, China*

[2]*Sai Ling Pharmaceutical Technology Group Co., Ltd, Yuxi City, China*

[3]*Research Program in Systems Oncology, Faculty of Medicine, University of Helsinki, Helsinki, 00290, Finland*

[*]*Corresponding author, Min Li: limin@mail.csu.edu.cn*

Contents

S1 Distribution of Dataset Analysis **2**

S2 MolDBG Pre-training and Fine-tuning Paradigm **3**

S3 Model Hyperparameters **5**

S4 Evaluation Metrics **6**

S5 Cold-Start Generalization Evaluation **7**

S6 Cryptic Pocket Protein Binding Site Prediction **8**

S7 Structural Novelty Analysis of Generated Molecules **10**

S8 LSQM Module Hyperparameter Ablation Experiments **13**

S1. Distribution of Dataset Analysis

We analyzed the statistical characteristics of the BioLip-BS datasets to determine the hyperparameters of the Binary Cross-Entropy (BCE) loss for binding site prediction. The detailed statistics are summarized in Table S1. Specifically, the BioLip-BS dataset comprises 2,993,769 amino acid residues, of which 121,487 are annotated as binding sites (4.06%). Furthermore, we visualized the density distributions of protein sequence lengths, molecular lengths, and affinity values for both the pre-training and fine-tuning datasets, as illustrated in Figure S1.

Table S1: Statistics of original datasets used in this study. Note: "/" indicates that the dataset does not contain binding site labels.

Dataset	Proteins	Drugs	Interaction	Binding sites (%)
BindingDB	11,382	1,390,080	3,173,561	/
Q-BioLiP	154,282	45,237	488,259	-
BioLip-BS	6,408	3,782	11,045	4.06%

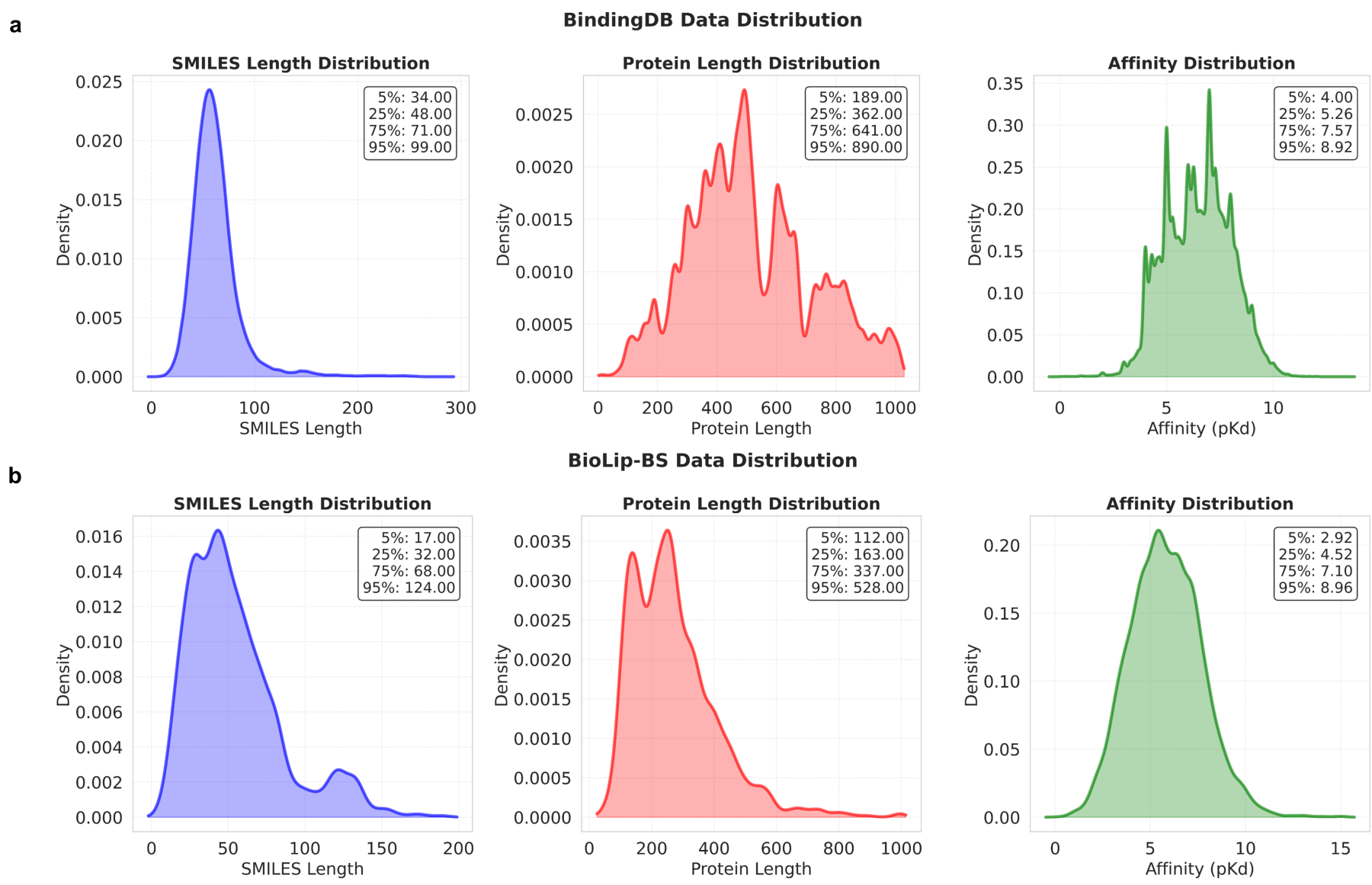

Figure S1: Distribution of dataset properties. The top row displays the density distributions of molecular length, protein sequence length, and affinity scores for the BindingDB dataset. The bottom row presents the corresponding distributions for the BioLip-BS dataset. For BindingDB, the IC_{50} metric was utilized. To ensure consistency in visualization, all affinity values were transformed into their negative logarithmic forms (pK_d or pIC_{50}).

S2. MolDBG Pre-training and Fine-tuning Paradigm

We hereby provide a supplementary description of the two-stage pre-training and fine-tuning paradigm, as illustrated in Figure S2.

Given the scarcity of data in the BioLip-BS dataset, training a stable, high-performance molecular design model from scratch is challenging. To address this, we implemented a pre-training phase for the MolDBG architecture using the BindingDB dataset, initially guided by the dual tasks of molecular design and affinity prediction. During pre-training, we employed ESM-2 and MolFormer to extract token-wise representations for proteins and molecular SMILES, respectively. Concurrently, a DTA-based conditional sampler was used to generate pseudo-ligand representations, which were concatenated along the batch dimension to construct multi-task inputs. In this phase, the Latent Sites Query Module (LSQM) operates without an attention mask on the Key-Value (KV) pairs, thereby exposing all protein token representations for automated binding region analysis. Following standard procedures, the data passes through the LSQM and a multi-layer Transformer encoder to learn interaction representations. Subsequently, the model is trained jointly using only the DTA-Decoder and the conditional Mol-Decoder. Based on prior research on attention visualization, we posit that the attention mechanism in the LSQM can theoretically learn to autonomously attend to the actual binding residue locations.

During the fine-tuning phase, the processes of standard token extraction, interaction token querying, and interaction representation fusion remain consistent with the pre-training phase. However, we introduced two critical modifications during fine-tuning. First, we implemented a masking strategy for the KV tensors in the LSQM, enabling MolDBG to mine binding sites within specified regions. To prevent posterior collapse, we adopted a simulated annealing-inspired approach, designing a linear masking schedule. Within a single batch, the input exposure is gradually adjusted from full-protein accessibility for all samples to a mixed-exposure mode (in which some samples remain fully exposed while others have non-binding-region tokens masked). To prevent label leakage, we introduced randomness into the open regions via random-length window convolution, random offsets, and the additional random exposure of a fixed proportion of tokens. Second, we incorporated the Binding Sites Decoder, modeling it directly on the LSQM's query outputs. Leveraging the inductive bias established in the pre-trained LSQM, we jointly optimized all three tasks using explicit gradient guidance from the binding site prediction task. This strategy enforces a hierarchical learning paradigm, compelling the model to extract latent binding-residue tokens before performing cross-modal self-attention for interaction representation learning.

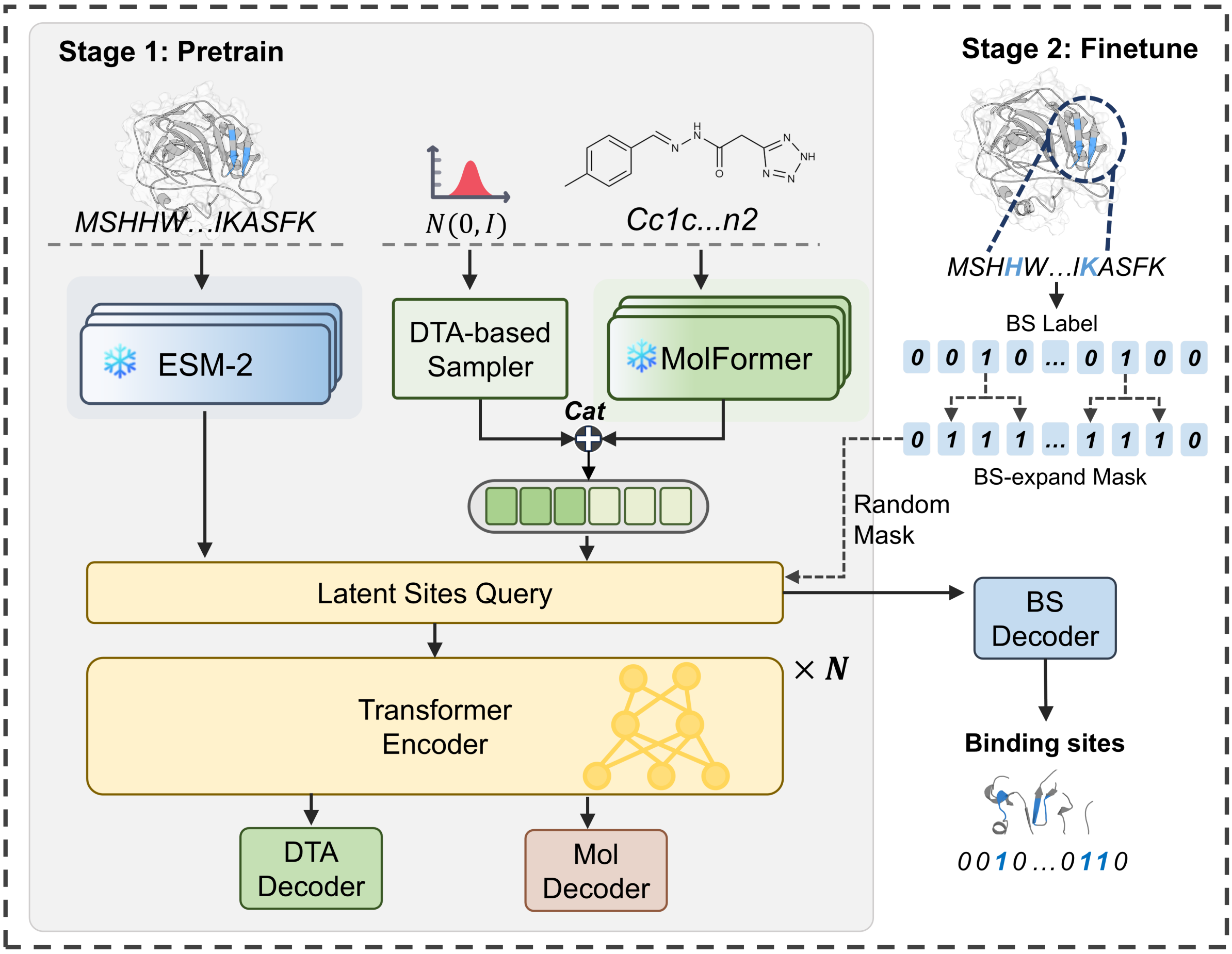

Figure S2: MolDBG Pre-training-Fine-tuning Framework Diagram

S3. Model Hyperparameters

We report the hyperparameter settings for each module of MolDBG in the Table S2 and Table S3.

Table S2: Hyperparameter settings of the DTI module.

Parameter	Value
Maximum protein sequence length	1024
Maximum molecule sequence length	256
Protein random mask ratio	0.15
LSQM minimum window size	5
LSQM maximum window size	25
Ratio of extra open tokens (binding sites)	0.1
Number of Encoder layers	4
Number of Encoder attention heads	16
Number of LSQM layers	2
Number of LSQM attention heads	32
Dropout rate	0.1
Hidden dimension size	768

Table S3: Detailed hyperparameter settings for Multiple Decoder.

Hyperparameter	Value
Model dimension (d)	768
DTA Decoder attention heads	16
Number of experts (N_e)	8
Top-k selection	2
Auxiliary loss weight (λ)	0.01
Hidden dimension	768
BS Decoder attention heads	16
Convolution kernel size	5
Convolution padding	2
Feed-forward network dimension	$4 \times d$
Mol Decoder attention heads	8
Dropout rate	0.1
Conditional MLP hidden dimension (D)	128

S4. Evaluation Metrics

For the intrinsically disordered proteins and cryptic pocket proteins, annotation labels are typically defined at the pocket or regional level. Since ground-truth annotations often cover broader regions than the specific binding residues of a single ligand, traditional exact-match metrics may underestimate the model's utility. To accurately evaluate performance against these targets, we defined a Precision-based Success Rate (SR) that incorporates a sequence-based tolerance mechanism. A sample is considered a success if the precision of its predicted binding sites exceeds a threshold δ:

$$SR = \frac{1}{M} \sum_{j=1}^{M} \mathbb{I}(\text{Precision}_j > \delta) \tag{S1}$$

where M is the total number of samples. For the j-th sample, let $\mathcal{G}_j$ be the set of ground truth residue indices and $\mathcal{P}_j$ be the set of predicted residues. To account for the local conformational flexibility of protein side chains, we introduced a tolerance parameter k. A predicted residue $p \in \mathcal{P}_j$ is considered a valid hit if it matches any ground truth residue within a sequence distance of k.

The precision is thus defined as:

$$\text{Precision}_j = \frac{\sum_{p \in \mathcal{P}_j} \mathbb{I}(\exists g \in \mathcal{G}_j, |p - g| \leq k)}{|\mathcal{P}_j|} \tag{S2}$$

In our experiments, we conducted a multi-level robustness evaluation by varying the success threshold δ (e.g., $\{0.3, 0.5, 0.7, 0.9\}$) and the tolerance k (e.g., $\{0, 1, 2, 3, 4\}$), where $k = 0$ corresponds to the strict exact match. We note that k is a sequence-distance tolerance and does not assert spatial proximity; we therefore additionally report the strict exact-match results ($k = 0$) for all dynamic-target benchmarks. We further emphasize that the UniProtKB-IDP and PocketMiner benchmarks provide only binding-site/pocket annotations without experimental affinity labels; consequently, affinity metrics (MSE/Pearson/CI) cannot be computed on these datasets.

S5. Cold-Start Generalization Evaluation

To rigorously evaluate the cold-start generalization capability of MolDBG in realistic drug discovery scenarios, we performed a strict sequence-identity-based cold-start evaluation on the BioLip-BS dataset. First, instead of the original partitioning protocol, we used MMseqs2 to perform comprehensive pairwise sequence alignments across all proteins in BioLip-BS. To prevent data leakage from homologous proteins, we applied a strict threshold of ¡30% sequence identity for clustering. Second, the BioLip-BS dataset was clustered and partitioned into training, validation, and test sets in an 8:1:1 ratio based on the MMseqs2 clustering results. This guarantees that no protein in the validation or test set shares $\geq 30\%$ sequence identity with any protein in the training set. The validation set was used exclusively to monitor training and select the best model checkpoint, while the test set served as the protein cold-start subset. In parallel, we constructed a SMILES cold-start subset by filtering standardized SMILES sequences from the protein cold-start subset. To establish a competitive baseline for this challenging scenario, we retrained and evaluated a representative deep learning model, DTIAM, under the identical splitting strategy. As shown in Table S4, we report a comprehensive performance comparison between MolDBG and DTIAM across five core affinity metrics: mean squared error (MSE), root mean squared error (RMSE), Pearson correlation coefficient (r), Spearman rank correlation coefficient (ρ), and concordance index (CI). MolDBG consistently outperforms DTIAM across the ranking and correlation metrics (Pearson, Spearman, and CI) while achieving comparable regression error (MSE/RMSE) under this strict low-homology constraint, demonstrating that MolDBG generalizes to unseen target proteins and ligands by capturing structural binding patterns rather than relying on sequence memorization.

Table S4: Cold-start generalization performance comparison between DTIAM and MolDBG.

**Model**	**Cold Start**	**MSE**	**RMSE**	**Pearson (r)**	**Spearman (ρ)**	**CI**
DTIAM	Protein	2.2704	1.5068	0.5844	0.5850	0.7053
	Molecule	1.9562	1.3986	0.5551	0.5541	0.6940
MolDBG	Protein	2.2842	1.5114	0.6067	0.6026	0.7125
	Molecule	1.9569	1.3989	0.5996	0.6001	0.7121

S6. Cryptic Pocket Protein Binding Site Prediction

In Table S5, we present detailed specifications of the PocketMiner dataset, including PDB identifiers, corresponding chain information, total protein residue counts, and residue counts for the cryptic pockets. We also report the number of effective residue hits achieved by MolDBG for these cryptic pocket proteins. Furthermore, we evaluate performance using the SR metric, which incorporates a sequence-distance tolerance mechanism. The results displayed in the table correspond to a sequence-distance tolerance of zero ($k = 0$) across varying thresholds, where '1' denotes a successful identification and '0' denotes failure.

Table S5: PocketMiner test results. Detailed prediction results on Apo protein structures with different SR thresholds

Apo information				BS prediction	SR			
PDB ID	Chain	Residues	Pocket Nums	Valid Hits	$\delta_{0.3}$	$\delta_{0.5}$	$\delta_{0.7}$	$\delta_{0.9}$
1EZM	A	298	21	14	1	1	1	1
1J8F	C	303	26	7	1	0	0	0
1KMO	A	661	22	3	1	0	0	0
1KX9	B	102	32	14	1	1	1	1
1S2O	A	244	14	1	1	0	0	0
1TVQ	A	125	35	15	1	1	1	1
1URP	A	271	13	11	1	1	1	1
1Y1A	A	183	7	0	0	0	0	0
2CEY	A	306	18	12	1	1	1	1
2FJY	A	136	19	1	1	1	1	1
2HQ8	B	182	25	3	1	1	1	0
2LAO	A	238	15	13	1	1	1	1
2OY4	A	157	26	10	1	1	1	1
2W9T	A	159	40	23	1	1	1	1
2ZKU	B	561	31	8	1	0	0	0
3FVJ	A	124	21	7	1	1	0	0
3KJE	A	254	21	9	1	1	1	0
3NX1	A	162	11	1	0	0	0	0
3P53	A	486	16	12	1	1	1	0
3PPN	A	270	12	9	1	1	1	0
3QXW	B	124	20	18	1	1	1	1
3UGK	A	372	42	11	1	1	1	1
4I92	A	289	19	8	1	1	1	1
4IC4	A	372	30	1	1	0	0	0
4P0I	B	256	21	15	1	1	1	1
4R72	A	318	24	17	1	1	1	1
4V38	A	388	23	7	1	1	1	0
4W51	A	164	17	9	1	1	1	0
5G1M	A	333	19	6	1	1	0	0
5H9A	A	139	22	10	1	1	0	0
5NIA	A	609	27	17	1	1	1	0
5NZM	A	483	27	2	1	0	0	0
5UXA	A	301	43	5	1	1	1	1
5ZA4	A	488	24	15	1	1	1	1
6E5D	A	173	23	1	1	0	0	0
6HB0	A	292	15	11	1	1	1	1
6RVM	C	308	26	18	1	0	0	0
6YPK	A	327	24	14	1	1	1	0

S7. Structural Novelty Analysis of Generated Molecules

To verify that MolDBG performs genuine de novo design rather than memorizing training molecules, we quantified the structural similarity between the generated molecules and the BioLip-BS dataset. Specifically, we used MolDBG to generate a library of 10,000 molecules for each target protein (EGFR and VEGFR2) under a given affinity value. For each generated molecule, we computed its maximum Tanimoto similarity (T_{max}) to the entire deduplicated, canonicalized BioLip-BS training set using ECFP4 fingerprints (Morgan, radius = 2, 2048 bits).

Table S6: Structural Novelty Statistics (excluding molecules with $T_{\text{max}} = 1.0$)

threshold	EGFR	VEGFR2
Median T_{max}	0.4430	0.4725
$T_{max} > 0.40$	58.94%	68.95%
$T_{max} > 0.60$	30.44%	24.90%
$T_{max} > 0.85$	5.60%	1.90%

As shown in Figure S3 and Table S6, we present the distribution and cumulative distribution of T_{max}, together with similarity-ratio statistics for the molecules generated under different target affinity conditions. The overwhelming majority of generated molecules fall well below the near-duplicate threshold (the median T_{max} lies in the low range, with a negligible fraction exceeding $T_{max} = 0.85$). In contrast, the internal diversity of the generated set remains high. It confirms that the conservative Novelty score reflects the intended constraints of conditioning on specific target sequences and affinity values, rather than memorization of training molecules. We additionally provide part of the affinity-conditional molecular design results for the EGFR and VEGFR2 targets in Figures S5 and S6.

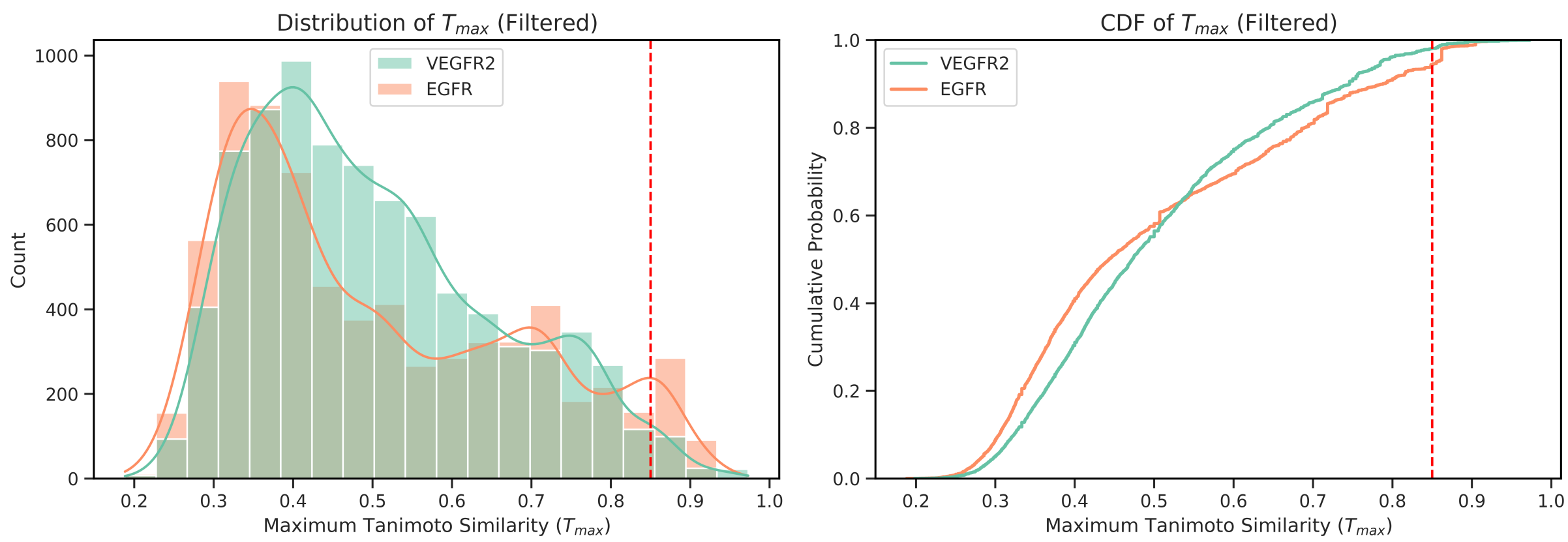

Figure S3: The distribution (left), cumulative distribution (right) of T_{max}.

In addition, for the six conditionally generated candidates highlighted in Figure 5 of the main text (the top three for EGFR and VEGFR2, respectively), we provide a

2D structural comparison against their closest training-set analog, reporting the pairwise Tanimoto similarity and Bemis-Murcko scaffold agreement for each candidate. As shown in Figure S4, these candidates exhibit low similarity to their nearest training neighbors and frequently differ in scaffold, providing direct quantitative evidence that they are genuinely novel hits rather than retrieved training molecules. Although the generated molecules share scaffolds with the training set, we attribute this to the model legitimately drawing on high-affinity scaffolds in the training data for molecular design under multiple constraints. In summary, these results demonstrate that our model achieves competitive performance in conditional molecular design.

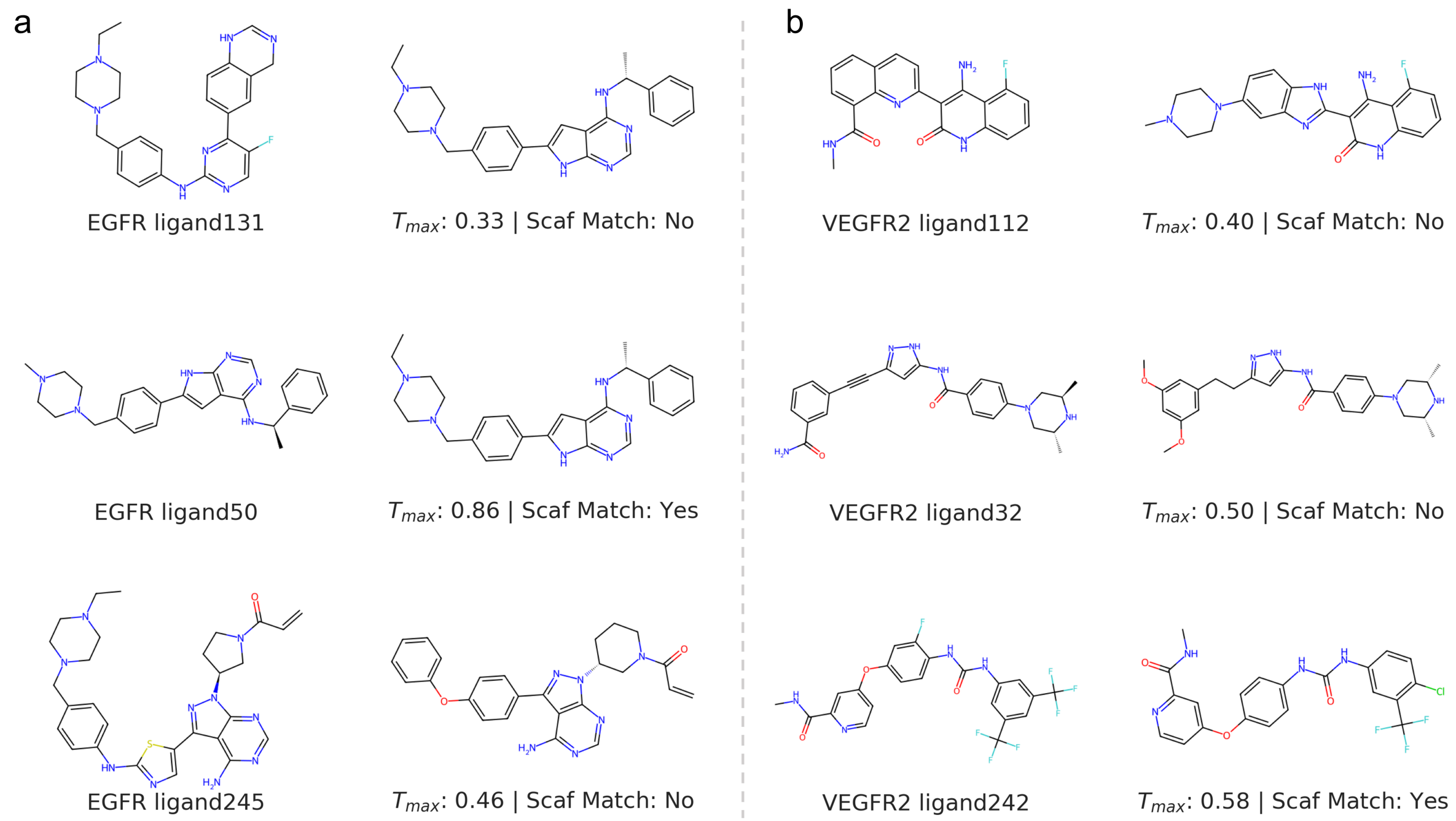

Figure S4: EGFR (a) and VEGFR2(b) target generation molecule compare with the closest train molecule.

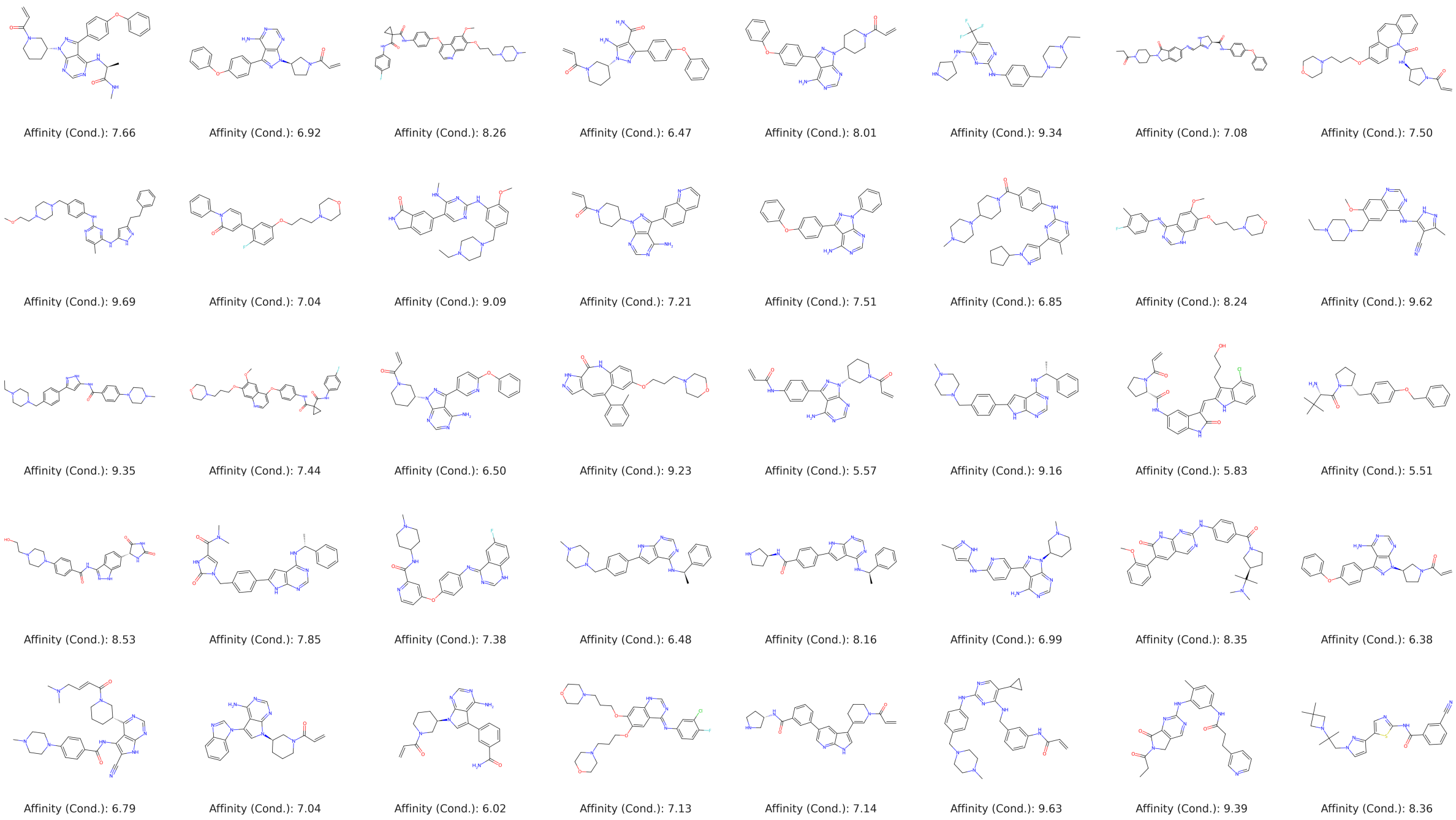

Figure S5: The affinity-conditional molecule design result for target EGFR.

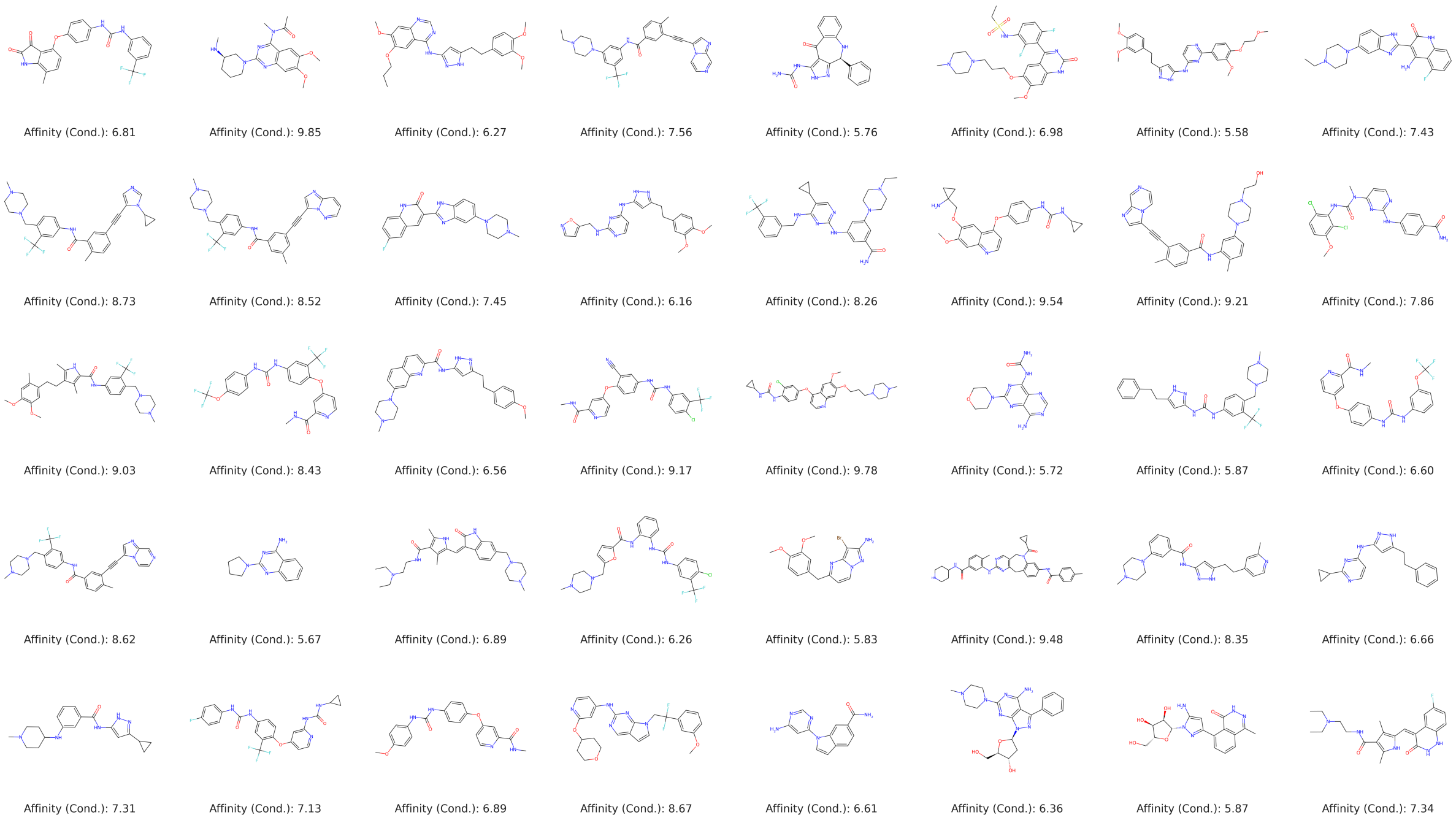

Figure S6: The affinity-conditional molecule design result for target VEGFR2.

S8. LSQM Module Hyperparameter Ablation Experiments

In this section, we conducted a comprehensive hyperparameter analysis of the Latent Sites Query Module (LSQM). By modulating the number of intermediate layers and attention heads, we assessed the impact of these parameters on the model's capacity to extract latent protein binding residues and learn drug-target interactions. The results are detailed in Table S7, S8, S9 and S10. It should be noted that, unlike the main-text results (which are averaged over 5-fold cross-validation), the ablation results reported in this section were obtained from a single independent run for each configuration; consequently, minor numerical differences from the corresponding main-text values are expected and do not indicate any inconsistency.

Table S7: The experimental results for MolDBG of affinity prediction task are the different numbers of LSQM query layers.

Setting	MSE	RMSE	Pearson	Spearman	r_m^2	CI
qlayer-1	0.7028	0.8383	0.8912	0.8924	0.7108	0.8788
qlayer-2	0.7033	0.8386	0.8906	0.8899	0.7165	0.8793
qlayer-4	0.6925	0.8322	0.8911	0.8913	0.7360	0.8776

Table S8: The experimental results for MolDBG of binding-sites prediction task are the different numbers of LSQM query layers.

Setting	F1	MCC	Recall	Acc	Precision	AUPRC	AUROC
qlayer-1	0.7920	0.7832	0.8104	0.9827	0.7744	0.8225	0.9683
qlayer-2	0.7835	0.7743	0.7993	0.9821	0.7683	0.8141	0.9720
qlayer-4	0.7765	0.7672	0.7993	0.9813	0.7551	0.8082	0.9734

Table S9: The experimental results for MolDBG of affinity prediction task are the different numbers of LSQM query heads.

Setting	MSE	RMSE	Pearson	Spearman	r_m^2	CI
qhead-8	0.7235	0.8506	0.8883	0.8914	0.7035	0.8792
qhead-16	0.7192	0.8481	0.8881	0.8870	0.7081	0.8755
qhead-24	0.7383	0.8593	0.8848	0.8871	0.7064	0.8768
qhead-32	0.7043	0.8392	0.8911	0.8917	0.7111	0.8790

Table S10: The experimental results for MolDBG of binding-sites prediction task are the different numbers of LSQM query heads.

Setting	F1	MCC	Recall	Acc	Precision	AUPRC	AUROC
qhead-8	0.7836	0.7744	0.7961	0.9822	0.7715	0.8169	0.9704
qhead-16	0.7774	0.7680	0.7897	0.9817	0.7655	0.8110	0.9721
qhead-24	0.7819	0.7727	0.7994	0.9819	0.7651	0.8173	0.9726
qhead-32	0.7809	0.7716	0.7951	0.9819	0.7671	0.8130	0.9712
